# Diversity, distribution and ecology of fungal communities present in Antarctic lake sediments uncovered by DNA metabarcoding

**DOI:** 10.1038/s41598-022-12290-6

**Published:** 2022-05-19

**Authors:** Láuren Machado Drumond de Souza, Juan Manuel Lirio, Silvia Herminda Coria, Fabyano Alvares Cardoso Lopes, Peter Convey, Micheline Carvalho-Silva, Fábio Soares de Oliveira, Carlos Augusto Rosa, Paulo Eduardo Aguiar Saraiva Câmara, Luiz Henrique Rosa

**Affiliations:** 1 Laboratório de Microbiologia Polar e Conexões Tropicais, Departamento de Microbiologia, Instituto de Ciências Biológicas, Universidade Federal de Minas Gerais, P. O. Box 486, Belo Horizonte, MG 31270-901 Brazil; 2 Instituto Antártico Argentino, Buenos Aires, Argentina; 3 Laboratório de Microbiologia, Universidade Federal do Tocantins, Porto Nacional, Brasil; 4 British Antarctic Survey, NERC, High Cross, Madingley Road, Cambridge, CB3 0ET UK; 5 Department of Zoology, University of Johannesburg, PO Box 524, Auckland Park, 2006 South Africa; 6 Departamento de Botânica, Universidade de Brasília, Brasília, Brasil; 7 Departamento de Geografia, Universidade Federal de Minas, Gerais, Belo Horizonte, MG Brasil

**Keywords:** Microbial communities, Environmental microbiology, Fungi

## Abstract

We assessed fungal diversity in sediments obtained from four lakes in the South Shetland Islands and James Ross Island, Antarctica, using DNA metabarcoding. We detected 218 amplicon sequence variants (ASVs) dominated by the phyla *Ascomycota*, *Basidiomycota*, *Mortierellomycota*, *Mucoromycota* and *Chytridiomycota*. In addition, the rare phyla *Aphelidiomycota*, *Basidiobolomycota*, *Blastocladiomycota*, *Monoblepharomycota*, *Rozellomycota* and *Zoopagomycota* as well as fungal-like Straminopila belonging to the phyla *Bacillariophyta* and *Oomycota* were detected. The fungal assemblages were dominated by unknown fungal taxa (Fungal sp. 1 and Fungal sp. 2), followed by *Talaromyces rubicundus* and *Dactylonectria anthuriicola*. In general, they displayed high diversity, richness and moderate dominance. Sequences representing saprophytic, pathogenic and symbiotic fungi were detected, including the phytopathogenic fungus *D. anthuriicola* that was abundant, in the relatively young Soto Lake on Deception Island. The lake sediments studied contained the DNA of rich, diverse and complex fungal communities, including both fungi commonly reported in Antarctica and other taxa considered to be rare. However, as the study was based on the use of environmental DNA, which does not unequivocally confirm the presence of active or viable organisms, further studies using other approaches such as shotgun sequencing are required to elucidate the ecology of fungi in these Antarctic lake sediments.

## Introduction

Antarctica represents one of the most extreme regions of the planet, including polyextremophilic environments and habitats that combine cold, dry and ultra-oligotrophic conditions, and offers unique opportunities to discover and study extremophilic organisms^1^. The large majority of Antarctica (>99.6% of its area) is permanently covered by ice and snow. Nevertheless, terrestrial and freshwater ecosystems are present that provide habitats occupied by organisms living in some of the most extreme conditions on the planet^2^. Antarctic lakes offer multiple extreme environmental challenges, including low temperatures, high salinity, pH variation, seasonally high UV radiation and low nutrient availability^1^. Various types of lakes are present, of different size, depth, trophic status and age, whose ecosystems are dominated by microorganisms adapted to the extreme conditions^3^.

Lakes present in the maritime Antarctic are typically shallow systems (<10 m deep), transparent allowing penetration of high levels of light and UV radiation, with low temperatures and low available nutrients^4^. However, the complex geology of the different islands in maritime Antarctica results in variable lake chemistry, which may favor the dominance of different microorganisms able to survive in these ecosystems and play a major role in the transfer of inorganic and organic material and energy^5^. The freshwater aquatic zones of Antarctica, including lakes, are in direct or indirect contact with air, rocks, soil and animals, and are bordered by vegetation; consequently, Antarctic lakes represent important sites for the study of microbial diversity and ecology^6^. Antarctic lake sediments can provide regional climatic archives and also shelter unique microbial communities, including bacteria, cyanobacteria, viruses, protists and fungi^7,8^.

Various Antarctic fungal communities have been characterized, including those in Antarctic lakes, which face various extreme conditions including low temperatures, high salinity, pH variation, seasonally high UV radiation and low nutrient availability^1^. To date, most fungal diversity studies in Antarctic lakes have applied traditional culturing methods^1,3,8–11^, which do not reveal the full diversity of the resident mycobiota. The recent application of approaches using metabarcoding have highlighted that fungal diversity in Antarctic lakes can potentially be considerably greater than previously appreciated^13–15^.

With this background, we hypothesized that metabarcoding approaches using high throughput sequencing (HTS) may detected cryptic fungal assemblages present in sediments of Antarctic lakes and contribute to better understanding of the complex resident fungal ecological networks involving saprophytic, mutualistic and parasitic taxa living under different extreme environmental conditions. We studied fungal richness, diversity and ecology in sediments sampled from four Antarctic lakes in the South Shetland Islands, north-west of the Antarctic Peninsula and James Ross Island to the east of the tip of the Antarctic Peninsula. We used a metabarcoding approach to assess and characterize fungal DNA sequence diversity present in sediment samples obtained from these four lakes.

## Methods

### Study sites and sediment sampling

**Figure 1:**
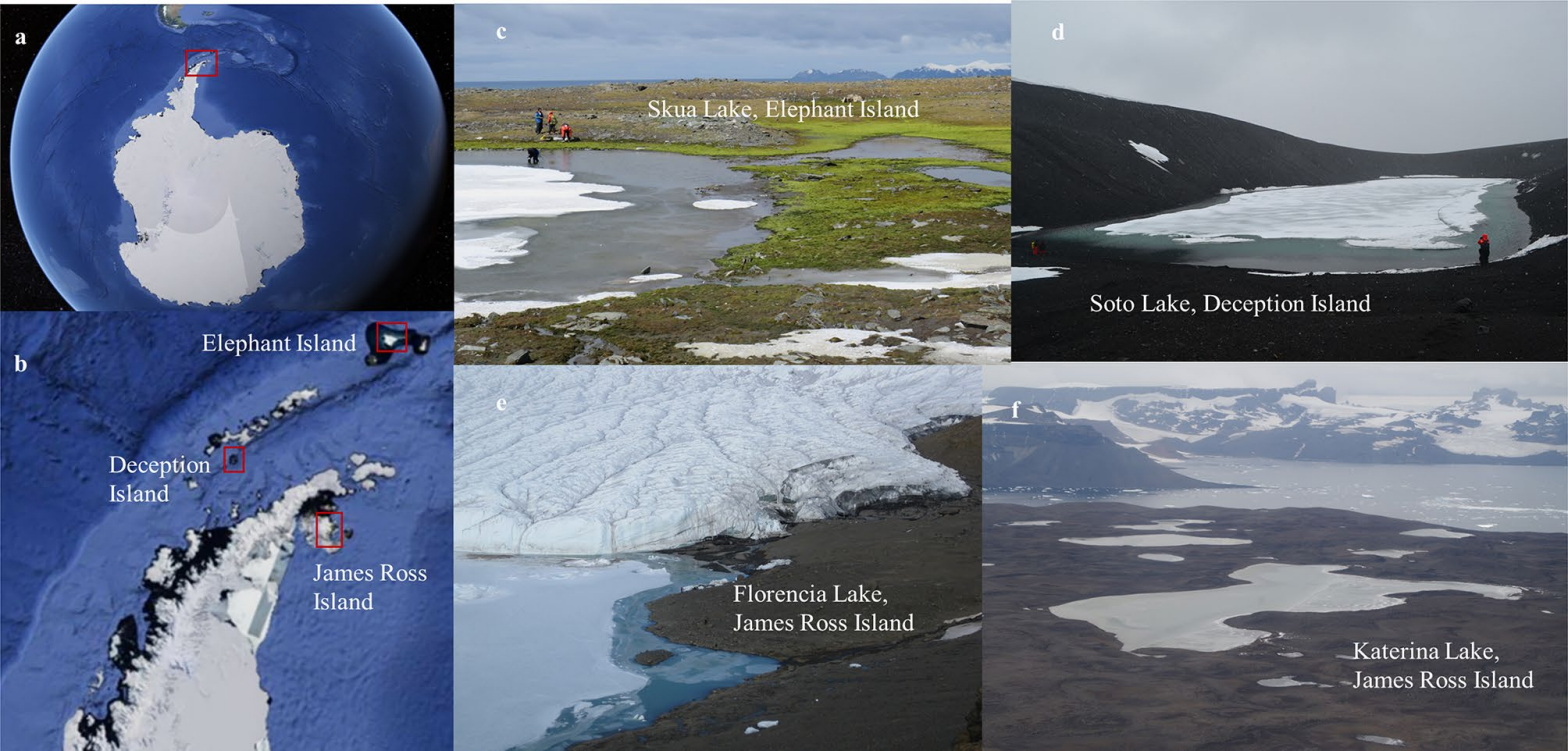
Satellite images (**a)** and (**b)** (obtained in Google Earth Pro, 2019). (**a**) Antarctica with the Antarctic Peninsula inside the red rectangle, (**b**) Elephant, Deception and James Ross Islands inside the red rectangle, (**c**) Skua Lake, Elephant Island (61°13′18.2″S; 55°21′54.3″W), (**d**) Soto Lake, Deception Island (62°58′44.6’’ S; 60°33′20.2’’ W), (**e**) Florencia (64°01′27.8’’ S; 57°40′00.6’’ W) and (**f**) Katerina Lakes (64°01′18.6’’ S; 57°43′26.2’’ W), James Ross Island. Photos (**c)** and (**e)** taken by Juan M. Lirio, photo (**f)** taken by Matej Roman and photo (**d**) taken by Luiz H. Rosa.

The four Antarctic lakes sampled were Skua Lake (Elephant Island), Soto Lake (Deception Island), both in the South Shetland Islands, and Florencia Lake and Katerina Lake (James Ross Island) (Fig. 1). Skua Lake is the northern-most of these lakes. It is an open basin, up to 1 m deep, fed by glacial meltwater from the Elephant Island Ice Cap. The lake occupies a depression on metamorphic rocks formed by glacial erosion. Soto Lake is a shallow crater depression formed by a phreatomagmatic eruption (maar). Its precise depth has not been measured, but is likely to be several meters. The lake is in a closed basin, fed by precipitation and its basement is formed by volcanic rock of basaltic composition. Florencia Lake is located on a sector of Clearwater Mesa known as Foreland on James Ross Island. It is an open basin, more than 10 m deep, directly abutting Blancmange Glacier. Its basement is formed by basal till deposits composed mainly of volcanic hyaloclastite breccias. Katerina Lake is located on top of Clearwater Mesa, lying on a relatively flat surface formed by a basaltic lava flow. It is an open basin, up to 1 m depth, and is fed by snowmelt. Each of these lakes displays different geological characteristics that may influence the resident mycobiota.

Sediments were collected from Skua Lake and Soto Lake during the austral summer of 2016/17 and from Katerina Lake and Florencia Lake in the austral summer of 2020/21 (Fig. 1; Table 1). Samples from all lakes were collected in triplicate using PVC pipes (60 mm diameter × 50 mm length), disinfected to avoid contamination as described by Ogaki et al.^8^. Sediments were sampled from three different points (each separated by approximately 50 m) in the littoral region of each lake at a depth of 20–50 cm. Approximately 500 g of each sediment sample was immediately sub-sampled, sealed, placed in sterile Whirl-pack bags (Nasco, Ft. Atkinson, WI) and frozen at −20 °C until processing in the laboratory at the Federal University of Minas Gerais, Brazil. There, the sampled was gradually thawed at 4 °C for 24 h before carrying out DNA extraction. In all DNA extraction steps, we proceeded under strict control conditions within a laminar flow hood to recover the fungal DNA and avoid contaminations.

**Table 1:** Lake locations, characteristics, sediment physicochemical data and diversity indices of fungal assemblages obtained from Skua Lake (Elephant Island), Soto Lake (Deception Island), Katerina Lake and Florencia Lake (James Ross Island).

Lake geological characteristics	Lake			
	Skua	Soto	Katerina	Florencia
Location	61° 13´ 18.2" S 55° 21´ 54.3" W	62° 59´ 05.4" S 60° 39´18.0" W	64° 01′ 25.5" S 57° 43′ 03.6" W	64° 01′ 24.0" S 57° 40′ 03.1" W
Altitude (meters above sea level)	59	13	250	25
Total area (m^2^)	6009	18,190	126,951	132,000
Perimeter (m)	400	630	2085	1854
Distance to coastline (m)	500	220	1,337	1,429
Depth (m)	0.4	> 3	1	> 10
Lake shape	Elongate (E–W)	Rectangular	Irregular	Circular
**Sediment chemical parameters**				
pH in H_2_O	6.1	8.8	8.4	8.5
Exchangeable P—mg dm^3–1^	218.4	19.5	120.5	9.3
Sum of exchangeable bases Ca + K + Mg (SB)—cmol_c_/dm^3^	0.73	3.51	5.53	7.59
Percentage of base saturation (PBS)—%	35.6	78.0	94.4	79.3
H + Al–potential acidity—cmol_c_ dm^3–1^	1.32	0.99	0.33	1.98
Cation exchange capacity at pH 7 (CEC)—cmol_c_ dm^3–1^	2.05	4.50	5.86	9.57
Total organic carbon (TOC)—dag/kg	0.07	0.08	0.54	0.38
Micronutrient Fe—mg dm^3–1^	84.5	231.2	252.2	44.0
Micronutrient Mn–mg dm^3–1^	2.3	11.1	54.2	63.2
**Resident fungal diversity indices**				
Number of DNA reads	33,429	71,983	54,939	46,813
Number of taxa	50	31	51	171
Fisher’s-α (diversity)	21.40	10.27	22.1	559.7
Margalef (richness)	9.25	5.66	9.44	32.09
Simpson's (dominance)	0.76	0.78	0.79	0.92

### Sediment chemical analysis

Sediment chemical analyses were performed following Embrapa^16^. pH was determined using a 1:2.5 sediment:deionized water ratio. Potential acidity (H + Al) was extracted with 0.5 mol L^−1^ Ca(OAc)_2_ buffered to pH 7.0 and quantified by titration with 0.0606 mol L^−1^ NaOH. Exchangeable Ca^2+^, Mg^2+^ and Al^3+^ were extracted with 1 mol L^−1^ KCl, and K^+^ and P^+^ were extracted with Melich^16^. The element levels in the extracts were determined by ICP (Al^3+^), flame emission (Na^+^, K^+^) and photocolorimetry (P) by the ascorbic acid method. Total organic carbon (TOC) was quantified by wet oxidation using the Walkley–Black method. All analyses were performed in triplicate. Total cation exchange capacity (CEC) was calculated as the sum of the bases (Ca^2+^, Mg^2+^, K^+^, Al^3+^) and potential acidity (H^+^ + Al^3+^). Chemical analyses of all sediment samples were performed in triplicate.

### DNA extraction, Illumina library construction and sequencing

Three replicate sub-samples were taken from the center of each core section under strict contamination control conditions. Total DNA was extracted from these using the FastDNA Spin Kit for Soil (MPBIO, Ohio, USA), following the manufacturer’s instructions. DNA quality was analyzed by agarose gel electrophoresis (1% agarose in 1 × Trisborate-EDTA) and then quantified using the Quanti-iT™ Pico Green dsDNA Assay (Invitrogen). The internal transcribed spacer 2 (ITS2) of the nuclear ribosomal DNA was used as a DNA barcode for molecular species identification of Fungi^17,18^ using the universal primers ITS3 and ITS4^19^. Library construction and DNA amplification were performed using the Herculase II Fusion DNA Polymerase Nextera XT Index Kit V2, following Illumina 16S Metagenomic Sequencing Library Preparation protocol (Part #15044223, Rev. B). Paired-end sequencing (2 × 300 bp) was performed on a MiSeq platform (Illumina) by Macrogen Inc. (South Korea). All quality control to avoid contamination of DNA extraction, PCR, sequencing were carried out and analyzed by Macrogen Inc.

### Data analysis and fungal identification

Quality analysis was carried out using BBDuk v. 38.87 in BBmap software^20^ with the following parameters: Illumina adapters removing (Illumina artefacts and the PhiX Control v3 Library); ktrim = l; k = 23; mink = 11; hdist = 1; minlen = 50; tpe; tbo; qtrim = rl; trimq = 20; ftm = 5; maq = 20. The remaining sequences were imported to QIIME2 version 2021.4 (https://qiime2.org/) for bioinformatics analyses^21^. The qiime2-dada2 plugin was used for filtering, dereplication, turn paired-end fastq files into merged, and remove chimeras, using default parameters^22^. Taxonomic assignments were determined for amplicon sequence variants (ASVs) in three steps. First, ASVs were classified using the qiime2-feature-classifier^23^ classify-sklearn against the UNITE Eukaryotes ITS database version 8.3^24^. Second, remaining unclassified ASVs were filtered and aligned against the filtered NCBI non-redundant nucleotide sequences (nt) database (October 2021) using BLASTn^25^ with default parameters; the nt database was filtered using the following keywords: “ITS1”, “ITS2”, “Internal transcribed spacer”, and “internal transcribed spacer”. Third, output files from BLASTn^25^ were imported to MEGAN6^26^ and taxonomic assignments were performed using the “megan-nucl-Jan2021.db” mapping file with default parameters and trained with Naive Bayes classifier and a confidence threshold of 98.5%. Taxonomic profiles were plotted using the Krona^27^. The heatmaps of ASV abundance and clustering analysis were created using Heatmapper^28^; clustering analysis was performed using the following parameters: Average Linkage, Spearman Rank Correlation and Z-score among samples for each ASV.

Many factors, including extraction, PCR and primer bias, can affect the number of reads obtained^29^, and thus lead to misinterpretation of absolute abundance^30^. However, Giner et al.^31^ concluded that such biases did not affect the proportionality between reads and cell abundance, implying that more reads are linked with higher abundance^32,33^. Therefore, for comparative purposes, we used the number of reads as a proxy for relative abundance. Fungal classification followed Kirk et al.^34^, Tedersoo et al.^35^, MycoBank (http://www.mycobank.org) and the Index Fungorum (http://www.indexfungorum.org).

### Fungal diversity and ecology

The relative abundances of the ASVs were used to quantify the fungal taxa present in the total sediments sampled, where fungal ASVs with relative abundance > 10% were considered dominant, those between 1 and 10% as intermediate and those with < 1% as minor (rare) components of the fungal community^36^. The numbers of reads were used to quantify taxon diversity, richness and dominance, using the following indices: (i) Fisher’s α, (ii) Margalef’s and (iii) Simpson’s, respectively. In addition, species accumulation curves were obtained using the Mao Tao index. All results were obtained with 95% confidence, and bootstrap values were calculated from 1000 replicates using the PAST computer program 1.90^37^. Functional assignments of fungal ASVs at species and generic levels were prepared using FunGuild^38^, which can be accessed at http://www.funguild.org/.

## Results

### Fungal taxonomy

A total of 207,164 DNA reads were detected in the sediments from the four lakes, representing 218 ASVs (see Supplementary Table S1 online). The Mao Tao rarefaction curves of the fungal assemblages detected in all four lake sediments are shown in Supplementary Fig. S1 online. The curves did not reach asymptote, indicating that further fungal sequence diversity is likely to be present in these sediments. Supplementary Figs. S2 and S3 show the abundances of fungal and allied organism ASVs at different hierarchical levels, which varied across the four lakes. Unknown fungi dominated the sequence assemblages, followed by the phyla *Ascomycota*, *Basidiomycota*, *Mortierellomycota*, *Mucoromycota* and *Chytridiomycota*. The lake sediment assemblages were dominated by Fungal sp. 1 (Skua, Katerina and Florencia Lakes), *Talaromyces rubicundus* (Soto and Katerina Lakes) and *Dactylonectria anthuriicola* (Soto Lake), in rank order, which displayed the highest relative abundances. However, richness (215 ASVs) of the fungal assemblages detected primarily comprised taxa of intermediate and rare relative abundance, including those of fungal phyla rarely (*Rozellomycota*, *Zoopagomycota*) or unreported (*Aphelidiomycota*, *Basidiobolomycota*, *Blastocladiomycota*, *Monoblepharomycota*) from Antarctica as well as members of the allied kingdom Straminopila (phylum *Oomycota*), which have also been infrequently detected in Antarctica in studies using traditional isolation methods. Ninety-five ASVs (47.57% of the total detected) could only be assigned to higher taxonomic levels (phylum, class, order or family) and may represent taxa not currently included in the available sequence databases or be new Antarctic records and/or previously undescribed taxa.

### Influence of physical and chemical properties of lacustrine sediments on the contained fungal communities

The fungal assemblages present in the lake sediments showed variation in their ecological diversity indices but, in general, despite the extreme physicochemical characteristics, they displayed high diversity and richness indices and moderate dominance (Table 1). The sediment fungal assemblage of Florencia Lake displayed the highest number of taxa and diversity indices, followed by those from Katerina, Skua and Soto Lakes.

**Figure 2:**
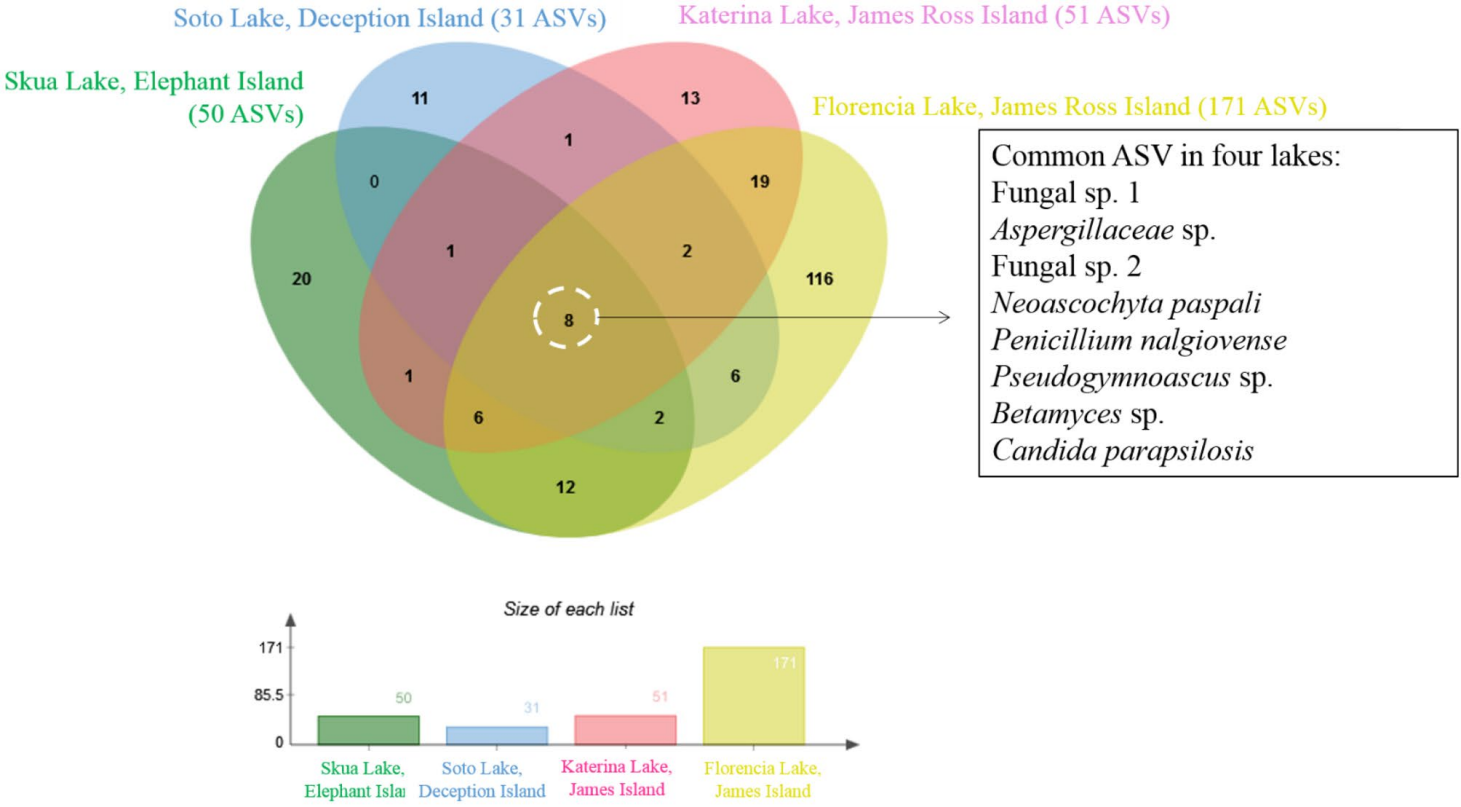
Venn diagram showing the distribution of fungal amplicon sequence variants (ASVs) among the sediment samples obtained from Skua Lake (Elephant Island), Soto Lake (Deception Island), Katerina Lake and Florencia Lake (James Ross Island).

The four lakes had sediments with similar chemical attributes, with low values for most of the elements. The sediment of Skua Lake differed from the other lakes in having slightly higher acidity (lower pH value and higher H + Al), higher exchangeable P content, and very low values for sum of bases and CEC. It was the only lake with dystrophic sediments (PBS < 50%). In contrast, sediments from Katerina Lake and Florencia Lake had the largest nutrient reserve in the form of exchangeable elements, mainly Ca, K and Mg, with the highest values of base saturation and CEC observed. The total organic carbon content was very low in sediments from all four lakes (< 1 dag kg^-1^). Of the total fungal ASVs detected, eight were present in all four lake sediments (Fig. 2; Supplementary Table S2). Taxa distribution varied across the four lakes, with sediments in each lake hosting some specific fungal taxa, in particular Florencia Lake with 116 fungal ASVs. The most abundant fungal ASV overall (Fungal sp. 1) was present in all four lake sediments. Beyond the eight cosmopolitan fungal ASVs that were shared amongst all lakes, no other ASVs were shared among Skua Lake (Elephant Island) and Soto Lake (Deception Island). Although both Katerina Lake and Florencia Lake are in relatively close proximity on Clearwater Mesa, James Ross Island (see Supplementary Fig. S4 online), the fungal assemblages detected in each (51 and 171 ASVs, respectively) shared only 19 fungal taxa and displayed distinct ecological diversity indices. Functional ecology assignments of the ASVs detected at generic level are shown in Supplementary Table S3 online, and indicated that the fungal communities present in the sediments of all four lakes were dominated by saprotrophic, plant and animal pathogenic and symbiotic taxa.

## Discussion

### Taxonomy and occurrence

The availability of studies of fungal diversity in Antarctic lake sediments has increased in recent years, but still remains limited^8,12^. Most previous studies of Antarctic lake fungal communities have used traditional culture-dependent methods^1,3,5,8,39^. However, recent applications of metabarcoding approaches have detected the presence of DNA sequences of different fungal taxa in Antarctic lake sediments^14,15^. The current study similarly confirmed the presence of high fungal sequence richness and diversity in sediments from four lakes sampled in the South Shetland Islands and James Ross Island. The study detected both the commonly encountered phyla *Ascomycota*, *Basidiomycota*, *Mortierellomycota*, *Mucoromycota*, *Chytridiomycota*, *Rozellomycota* and *Zoopagomycota* (cf. reports of da Silva et al.^40^, Ogaki et al.^14^ and Rosa et al.^15^), and the more rarely detected or unreported *Aphelidiomycota*, *Basidiobolomycota*, *Blastocladiomycota* and *Monoblepharomycota*, as well as members of the fungal-like kingdom Straminopila in the phylum *Oomycota*.

ASVs assigned to Fungal sp. 1, *T. rubicundus* and *D. anthuriicola* were the most abundant fungal taxa detected in the lake sediments. The most abundant ASV was Fungal sp. 1 (24.757%), suggesting a dominance of possibly undescribed fungi present in Antarctic lake sediment ecosystems. This unidentified dominant ASV provides an example of a sequence not currently included in the available sequence databases used in our study. *Talaromyces*, the teleomorphic form of *Penicillium*^41^, includes species pathogenic to humans, but also producers of antibiotics and enzymes potentially useful in biotechnological processes^42^. *Talaromyces rubicundus* has been reported to produce various extrolites such as islandicin chromophore, mitorubrin, mitorubrinic acid and rubratoxins^42^. In Antarctica, representatives of *Talaromyces* and *Penicillium* have been detected in different habitats and substrates including soil, marine and lake sediments, freshwater and seawater, plants, macroalgae, rocks, snow, ice^43^ and air^36^. *Talaromyces rubicundus* (anamorphic form of *Penicillium rubicundum*) was originally isolated from cultivated soil in Georgia, USA, and displays mesophilic characteristics and an optimal growth temperature of 25°C^44^. The genus *Dactylonectria* includes 14 species, some of which are recognized as soil-borne plant pathogens^45^. *Dactylonectria anthuriicola* (synonym of *Ilyonectria anthoriicola*) was isolated from the roots of *Anthurium* sp. in the Netherlands^46^. Recently, Parkinson et al.^47^ reported that *D. anthuriicola* caused significant root rot on avocado trees in Australia. Our study represents the first records of both *T. rubicundus* and *D. anthuriicola* in Antarctica.

Ninety-five ASVs, representing almost the half of the total community, were identified at phylum, class, order or family levels. These fungi may represent taxa not currently included in the available sequence databases or be undescribed taxa. However, as our study focused on fungal diversity assigned from environmental DNA samples, further specific taxonomic studies are required to elucidate if the apparently high richness of undescribed taxa does indeed represent previously unrecognized fungal species present in Antarctica.

### Diversity and ecology

Our fungal sequence diversity data can be compared with studies using both traditional culturing methods and the increasing number of recent metabarcoding studies of Antarctic lakes (see Supplementary Table S5 online). The diversity indices of fungal sequence assemblages detected in the sediments of the four lakes studied here were greater than those reported in culture-based studies but comparable with those using metabarcoding. Ogaki et al.^12^ used culture-dependent methods to characterize the same sediments from Skua and Soto Lakes, identifying only 13 and 6 fungal taxa, respectively, mostly at genus and species levels, contrasting with our data obtained using metabarcoding.

The four lakes also differed in their diversity indices, with Soto Lake and Florencia Lake displaying the lowest and highest diversity indices, respectively. No significant correlation was observed between the diversity indices and the sediment physicochemical parameters of the lakes. Considerable differences in the perimeters and depths of these two lakes may be correlated with their sediment accumulation and, hence, DNA deposition into the sediments, underlying the different levels of diversity detected.

Soto Lake is not surrounded by a well-developed community of mosses or lichens and is characterized by low nutrient availability. It represents an ecosystem still in formation and displayed the lowest diversity indices. Skua Lake showed intermediate diversity indices, which may be due to the rich terrestrial vegetation and presence of nesting skuas and giant petrels in its surroundings. Florencia Lake showed the highest diversity and richness indices, which were strongly divergent with those in Katerina Lake, despite their close proximity (separated by approximately 2050 m) (see Supplementary Fig. S4 online). However, these two lakes have geological differences and are surrounded by extensive moss and lichen communities. Florencia Lake has the greatest water inflow of the four studied lakes, being fed mainly by meltwater that carries significant quantities of clastic sediment, as well as receiving water from other lacustrine basin lakes with which it is connected. These are fed by snowmelt and are surrounded by a rich flora of mosses and microbial mats that provide organic matter to the lake. The local gradients in the Florencia Basin also encourage fluvial erosion of the moss carpets and the transport of fragments towards Florencia Lake. In contrast, Katerina Lake is fed by snowmelt and linked with other small lakes also fed by snowmelt through small ephemeral streams with low transport capacity, although it is also surrounded by a relatively rich terrestrial flora of mosses and lichens. The range of potential sources of organic matter input to Florencia Lake perhaps underlies this lake having much higher fungal diversity indices than Katerina Lake.

The chemical attributes of lake sediments reflect their geological contexts and the biological communities in their catchments. These attributes and the low TOC values suggest a mainly mineral composition of the sediments in the studied lakes. The lakes associated with basic igneous rocks, of basaltic composition (Soto, Katerina and Florencia) had higher SB values and other indices (PBS, CEC). These rocks contain minerals rich in Ca, K and Mg, which form a major constituent of the sediments in these lakes. Geochemical studies (personal communication Dr. Siliva Coria—Instituto Antártico Argentino) of Florencia Lake sediments have identified minerals such as plagioclase, potassium feldspar, piroxenes, zeolites and forsterite, that are rich in Ca, K, Na, Fe and Mg.

In contrast, Skua Lake is located in an area of metamorphic rocks that have greater content of aluminosilicate minerals, such as mica, quartz and garnet, with lower base content. Metamorphic rocks are commonly associated with more acidic and less fertile soils than rocks of basic composition^48^. Skua Lake is surrounded by rich vegetation and receives input of excrement from surrounding breeding birds, which leads to higher values of exchangeable P^49^. The sediment samples examined in this study were collected in the littoral regions of each lake at 20–50 cm depth, meaning that there could be a mix of present-day and past fungal communities dating from years to centuries ago contained in the sediments.

The fungal genera detected in the sediments display different ecological roles including saprophytes, mutualists, symbionts and parasites, as more generally reported for Antarctic fungi^43^. Among the genera with known ecological roles, saprophytes dominated the fungal communities present in all four lake sediments, followed by plant and animal pathogens and symbionts. Studies that have addressed the functional ecological roles of fungi in different Antarctic environments and habitats report saprophytes as the dominant functional group, followed by plant and animal pathogens and symbionts. The same functional ecological profile detected here has also been reported in studies sampling fungi in the air^36,50^, soil^51^, fresh water^13^ and rocks^52^ in Antarctica. Similar functional profiles were reported in metabarcoding studies of lake sediments reported by Ogaki et al.^14^ from Vega Island (located close to James Ross Island) and Rosa et al.^15^ in Hope Bay at the north-eastern tip of the Antarctic Peninsula. However, in sediment samples obtained from Soto Lake (Deception Island), the recognized phytopathogenic species *D. anthuriicola* dominated the sequence assemblage, although the species has not previously been recorded in Antarctica. Fungi inhabiting polar environments commonly display the capability to degrade organic matter at low temperature and release compounds containing carbon, nitrogen and other elements to other organisms^53^, suggesting that the saprophytic fungi detected in the four lake sediments studied here might host a complex fungal community that plays a vital role in the decomposition of organic matter under extreme conditions.

## Conclusions

Application of a metabarcoding approach revealed that sediments from the four lakes studied contained DNA potentially representing rich, diverse and complex fungal communities. These included both known fungi commonly reported in Antarctica (members of the phyla *Ascomycota*, *Basidiomycota*, *Mortierellomycota* and *Mucoromycota*), as well as others considered rare or not previously reported in Antarctica (*Chytridiomycota*, *Rozellomycota*, *Zoopagomycota*, *Aphelidiomycota*, *Basidiobolomycota*, *Blastocladiomycota*, *Monoblepharomycota*), and the presence of the fungal-like Straminopila. Antarctic lake sediments accumulate over long periods of time, and our data indicate that they may be considered a hotspot of fungal diversity, potentially including new and/or previously unreported species. The dominance of sequences of a small number of saprophytic and phytopathogenic taxa was notable. This metabarcoding study was based on the use of environmental DNA and further studies for instance using shotgun sequencing are now required to elucidate the ecology of Antarctic lake sediment fungi.

## Supplementary Information

Supplementary Information 1.

Supplementary Information 2.

Supplementary Information 3.

Supplementary Information 4.

Supplementary Information 5.

Supplementary Information 6.

Supplementary Information 7.

Supplementary Information 8.

## Data Availability

All data generated or analysed during this study are included in this published article and its supplementary information files.
