# Supplementary material for: Diversity, distribution and ecology of fungal communities present in Antarctic lake sediments uncovered by DNA metabarcoding
Source: Sci Rep. 2022 May 19;12:8407. doi: 10.1038/s41598-022-12290-6 (PMC9120451; doi:10.1038/s41598-022-12290-6)
Supplement: Supplementary file 1 — Supplementary Information 1. [file 41598_2022_12290_MOESM1_ESM.docx]

**Diversity, distribution and ecology of fungal communities present in Antarctic lake sediments uncovered by DNA metabarcoding**

Láuren Machado Drumond de Souza, Juan Manuel Lirio, Silvia H. Coria, Fabyano Alvares Cardoso Lopes, Peter Convey, Micheline Carvalho-Silva, Fábio Soares de Oliveira, Carlos Augusto Rosa, Paulo EAS Câmara and Luiz Henrique Rosa


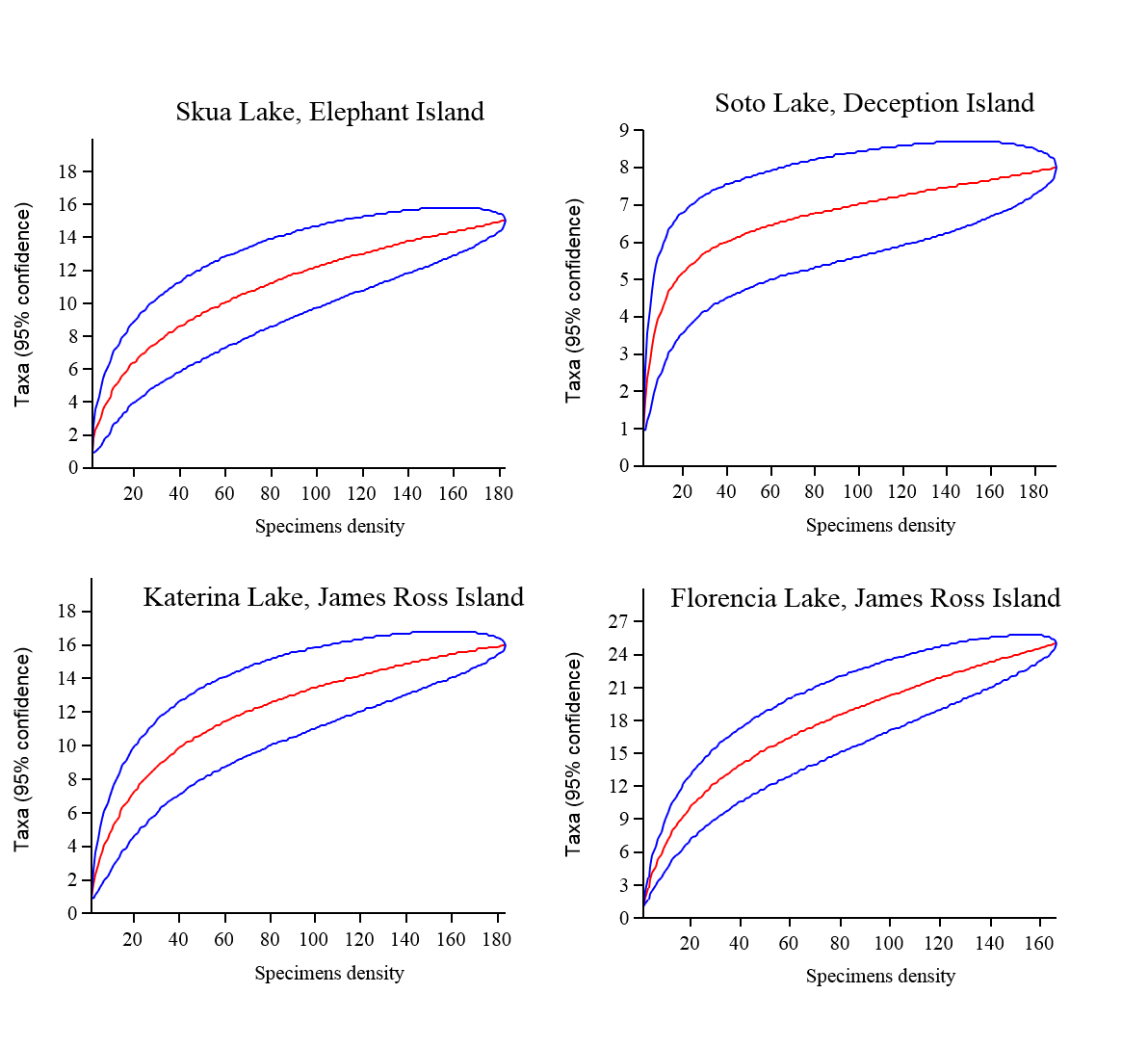


**Supplementary Figure S1.** Rarefaction curves, with 95% confidence limits, of fungal amplicon sequence variants (ASVs) obtained from the sediments of Skua Lake (Elephant Island), Soto Lake (Deception Island), Katerina Lake and Florencia Lake (James Ross Island).
