# Supplementary material for: Diversity, distribution and ecology of fungal communities present in Antarctic lake sediments uncovered by DNA metabarcoding
Source: Sci Rep. 2022 May 19;12:8407. doi: 10.1038/s41598-022-12290-6 (PMC9120451; doi:10.1038/s41598-022-12290-6)
Supplement: Supplementary file 2 — Supplementary Information 2. [file 41598_2022_12290_MOESM2_ESM.docx]

**Diversity, distribution and ecology of fungal communities present in Antarctic lake sediments uncovered by DNA metabarcoding**

Láuren Machado Drumond de Souza, Juan Manuel Lirio, Silvia H. Coria, Fabyano Alvares Cardoso Lopes, Peter Convey, Micheline Carvalho-Silva, Fábio Soares de Oliveira, Carlos Augusto Rosa, Paulo EAS Câmara and Luiz Henrique Rosa


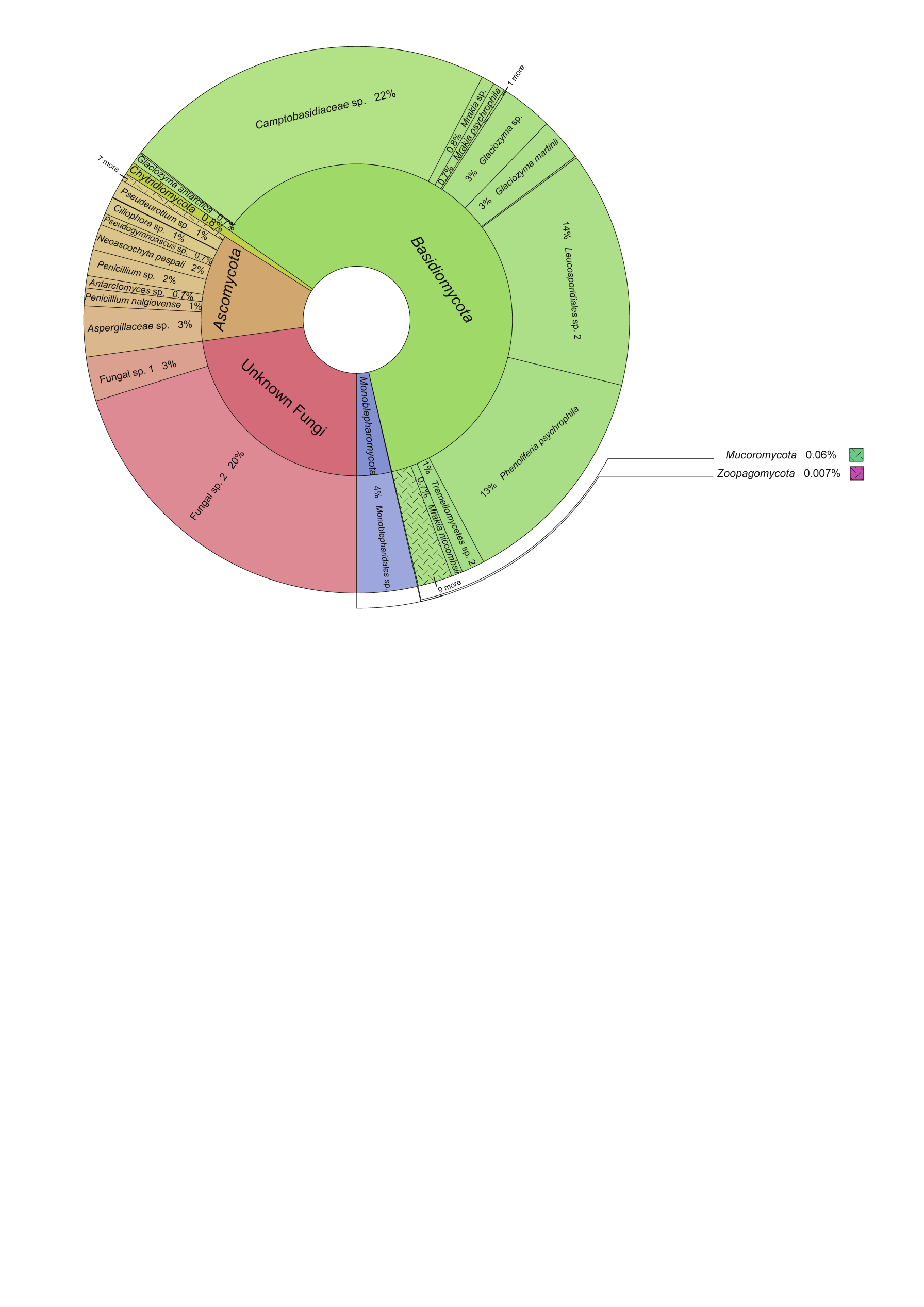


(**a**)


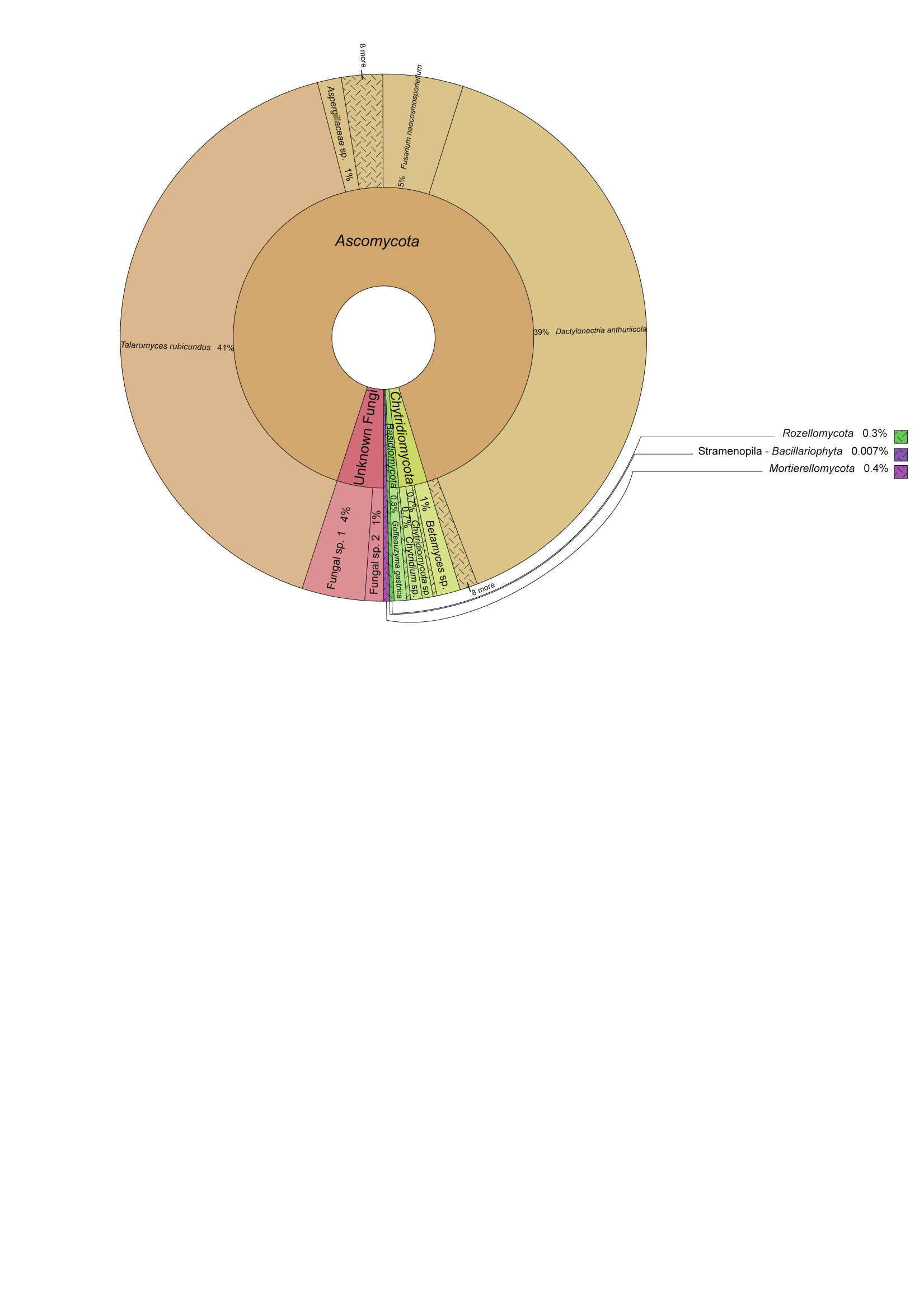


(**b**)


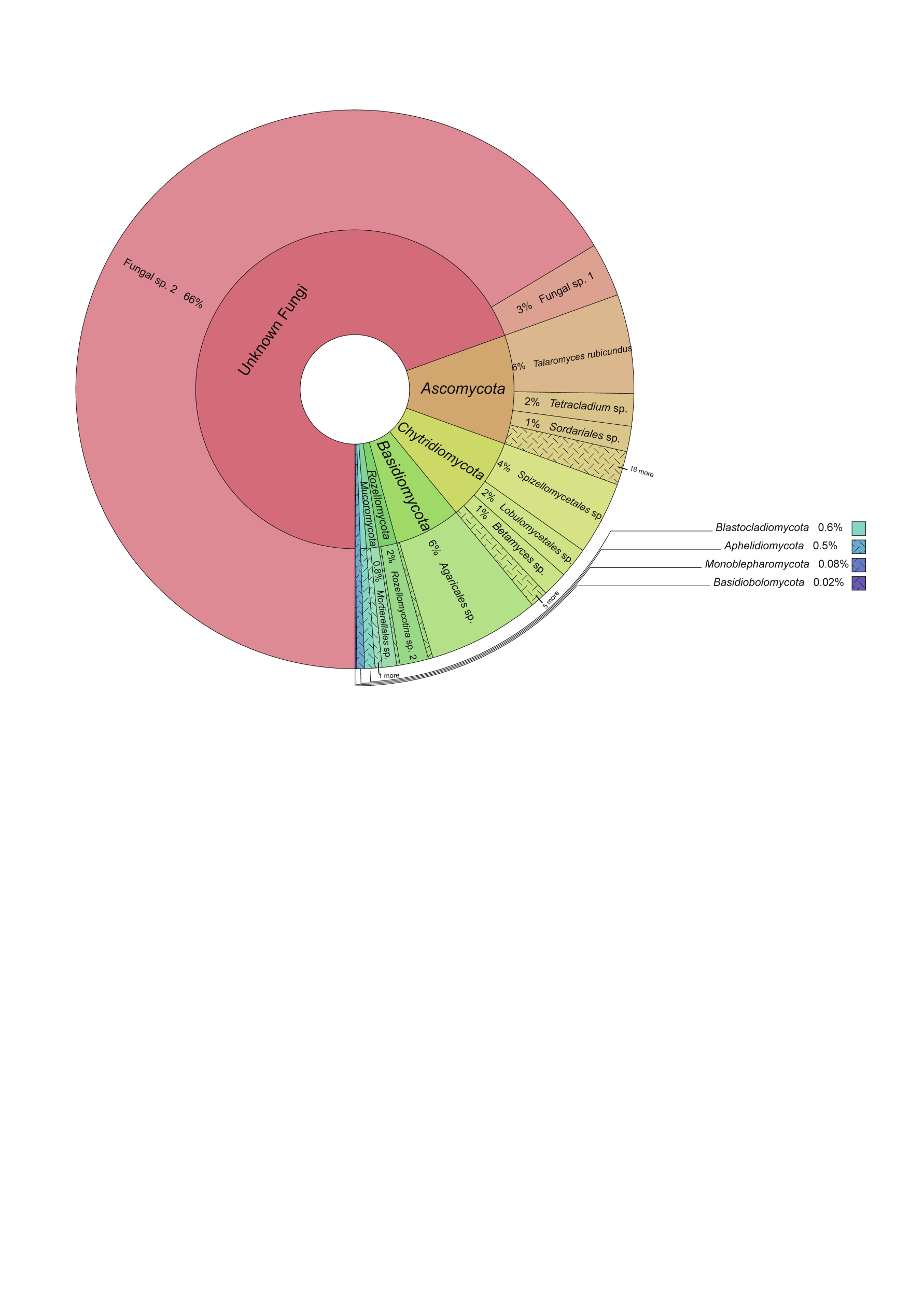


(**c**)


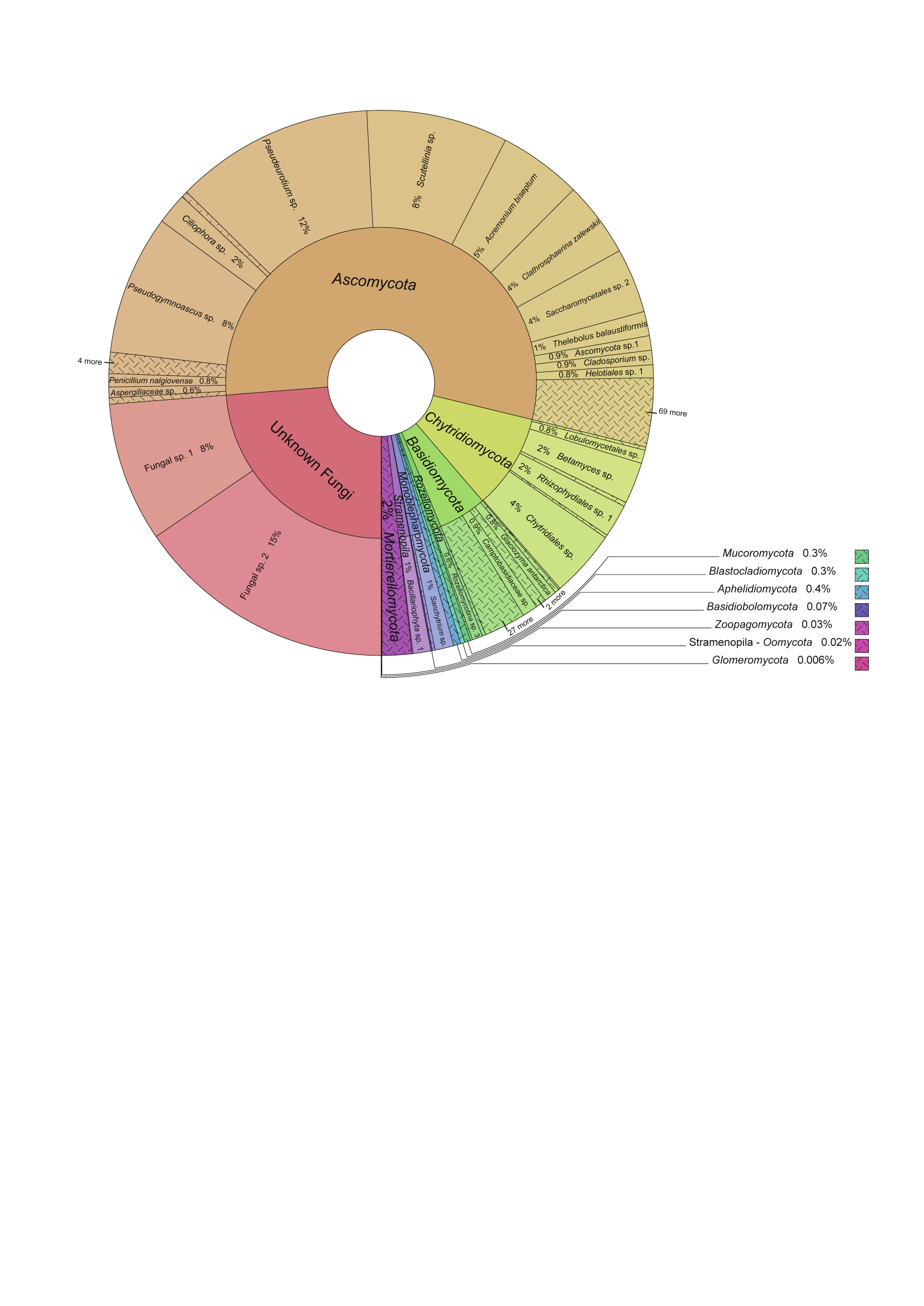


(**d**)

**Supplementary Fig. S2**. Krona chart showing the abundances of different fungal taxonomic levels detected in sediment samples from (**a**) Skua Lake (Elephant Island), (**b**) Soto Lake, Deception Island, (**c**) Katerina Lake, and (**d**) Florencia Lake, James Ross Island.
