# Supplementary material for: Diversity, distribution and ecology of fungal communities present in Antarctic lake sediments uncovered by DNA metabarcoding
Source: Sci Rep. 2022 May 19;12:8407. doi: 10.1038/s41598-022-12290-6 (PMC9120451; doi:10.1038/s41598-022-12290-6)
Supplement: Supplementary file 3 — Supplementary Information 3. [file 41598_2022_12290_MOESM3_ESM.docx]

**Diversity, distribution and ecology of fungal communities present in Antarctic lake sediments uncovered by DNA metabarcoding**

Láuren Machado Drumond de Souza, Juan Manuel Lirio, Silvia H. Coria, Fabyano Alvares Cardoso Lopes, Peter Convey, Micheline Carvalho-Silva, Fábio Soares de Oliveira, Carlos Augusto Rosa, Paulo EAS Câmara and Luiz Henrique Rosa


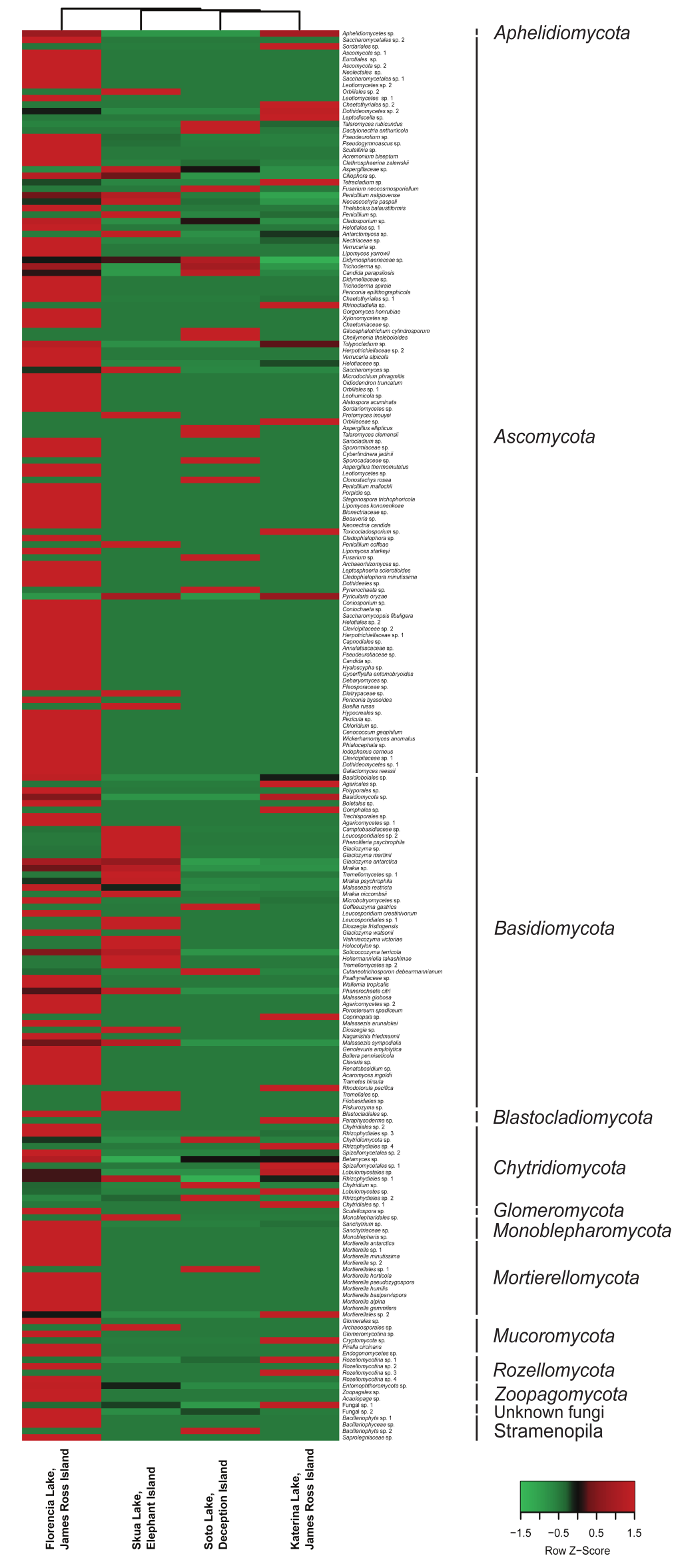


**Supplementary Fig. S3**. Heat map of fungal assemblage relative abundances. The color intensities range from red (highest relative abundance) to green (lowest relative abundance). These values represent percentages of DNA fungal reads in Skua Lake (Elephant Island), Soto Lake (Deception Island), Katerina Lake and Florencia Lake (James Ross Island). The heatmap of ASV abundance was performed using the following parameters: Average Linkage, Spearman Rank Correlation, and Z-score among samples for each ASV.
