# Supplementary material for: Diversity, distribution and ecology of fungal communities present in Antarctic lake sediments uncovered by DNA metabarcoding
Source: Sci Rep. 2022 May 19;12:8407. doi: 10.1038/s41598-022-12290-6 (PMC9120451; doi:10.1038/s41598-022-12290-6)
Supplement: Supplementary file 4 — Supplementary Information 4. [file 41598_2022_12290_MOESM4_ESM.docx]

**Diversity, distribution and ecology of fungal communities present in Antarctic lake sediments uncovered by DNA metabarcoding**

Láuren Machado Drumond de Souza, Juan Manuel Lirio, Silvia H. Coria, Fabyano Alvares Cardoso Lopes, Peter Convey, Micheline Carvalho-Silva, Fábio Soares de Oliveira, Carlos Augusto Rosa, Paulo EAS Câmara and Luiz Henrique Rosa


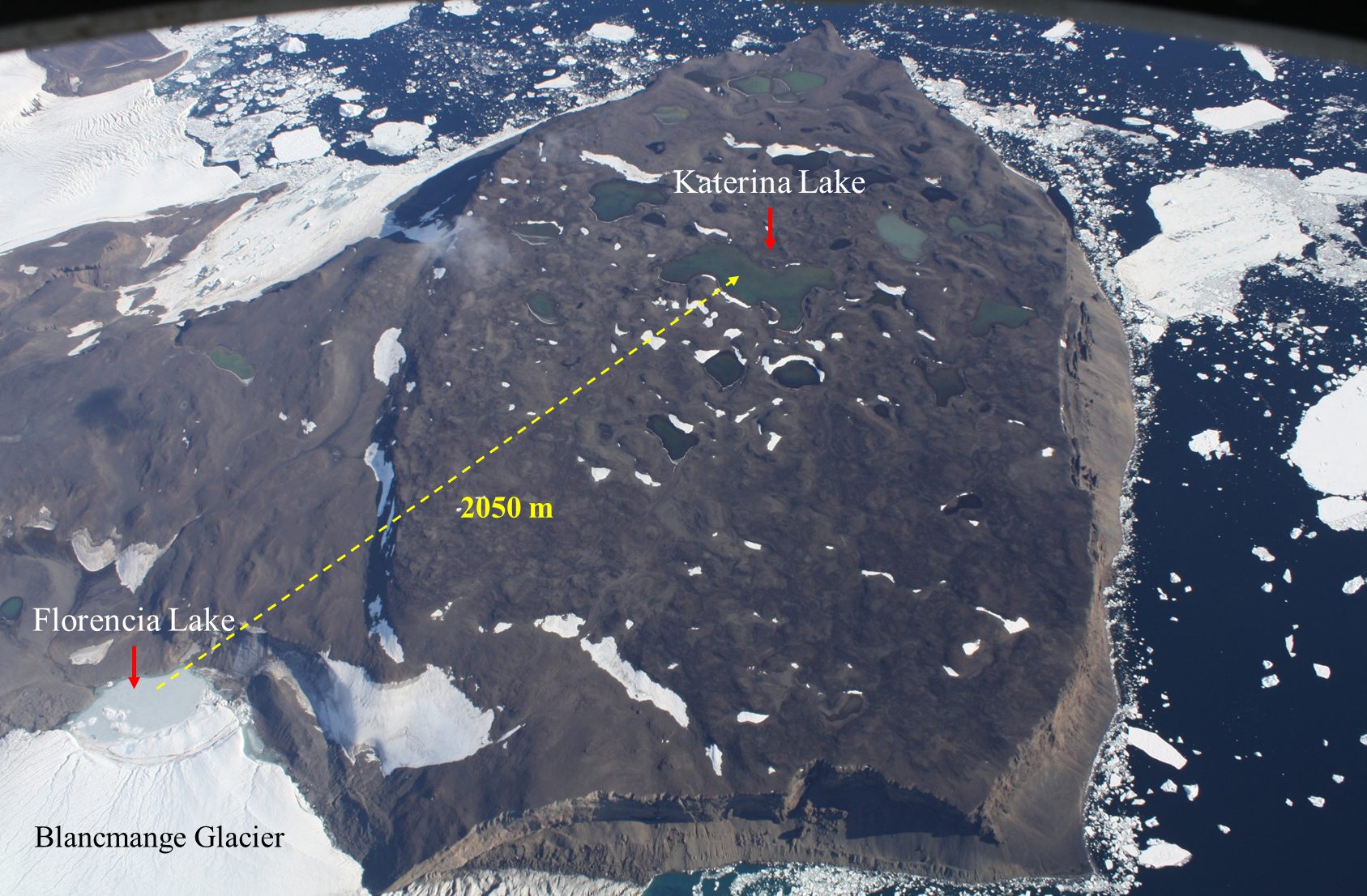


**Supplementary Figure S4**. Mesa Clearwater plateau showing the distance between Florencia and Katerina lakes at James Ross Island, Antarctica. Photo taken by Juan M. Lírio.
