# Supplementary material for: Diversity, distribution and ecology of fungal communities present in Antarctic lake sediments uncovered by DNA metabarcoding
Source: Sci Rep. 2022 May 19;12:8407. doi: 10.1038/s41598-022-12290-6 (PMC9120451; doi:10.1038/s41598-022-12290-6)
Supplement: Supplementary file 5 — Supplementary Information 5. [file 41598_2022_12290_MOESM5_ESM.docx]

**Diversity, distribution and ecology of fungal communities present in Antarctic lake sediments uncovered by DNA metabarcoding**

Láuren Machado Drumond de Souza, Juan Manuel Lirio, Silvia H. Coria, Fabyano Alvares Cardoso Lopes, Peter Convey, Micheline Carvalho-Silva, Fábio Soares de Oliveira, Carlos Augusto Rosa, Paulo EAS Câmara and Luiz Henrique Rosa

**Supplementary Table S1.** Relative abundances of the fungal amplicon sequence variants (ASVs) detected in lake sediment samples obtained from Skua Lake (Elephant Island), Soto Lake (Deception Island), Katerina Lake and Florencia Lake (James Ross Island).

|  |  |  |  | **Relative abundance (%) of fungal ASV in Lake/Island** | | | | |
| --- | --- | --- | --- | --- | --- | --- | --- | --- |
| **Source** | **Kingdom** | **Phylum** | **Fungal amplicon sequence variant** | **Skua/ Elephant** | **Soto/ Deception** | **Katerina/ James Ross** | **Florencia/ James Ross** | **Total** |
| UNITE | Fungi | *Ascomycota* | *Talaromyces rubicundus* | 0.000** | 40.961 | 5.735 | 0.000 | 15.754 |
|  |  |  | *Dactylonectria anthuriicola* | 0.000 | 39.311 | 0.000 | 0.017 | 13.663 |
|  |  |  | *Pseudeurotium* sp. | 1.130 | 0.000 | 0.000 | 11.814 | 2.852 |
|  |  |  | *Pseudogymnoascus* sp. | 0.694 | 0.207 | 0.049 | 8.302 | 2.073 |
|  |  |  | *Scutellinia* sp. | 0.000 | 0.000 | 0.000 | 8.380 | 1.894 |
|  |  |  | *Fusarium neocosmosporiellum* | 0.000 | 4.925 | 0.000 | 0.073 | 1.728 |
|  |  |  | *Aspergillaceae* sp. | 2.996 | 1.470 | 0.522 | 0.622 | 1.273 |
|  |  |  | *Clathrosphaerina zalewskii* | 0.000 | 0.390 | 0.000 | 4.478 | 1.148 |
|  |  |  | *Acremonium biseptum* | 0.000 | 0.000 | 0.000 | 4.928 | 1.113 |
|  |  |  | *Penicillium nalgiovense* | 1.051 | 0.529 | 0.365 | 0.790 | 0.629 |
|  |  |  | *Ciliophora* sp. | 1.008 | 0.000 | 0.043 | 1.854 | 0.593 |
|  |  |  | *Tetracladium* sp. | 0.000 | 0.000 | 1.873 | 0.358 | 0.578 |
|  |  |  | *Neoascochyta paspali* | 1.513 | 0.406 | 0.137 | 0.542 | 0.544 |
|  |  |  | *Thelebolus balaustiformis* | 0.000 | 0.000 | 0.000 | 1.437 | 0.325 |
|  |  |  | *Penicillium* sp. | 1.556 | 0.000 | 0.174 | 0.105 | 0.321 |
|  |  |  | *Cladosporium* sp. | 0.000 | 0.343 | 0.000 | 0.850 | 0.311 |
|  |  |  | *Antarctomyces* sp. | 0.730 | 0.000 | 0.194 | 0.080 | 0.187 |
|  |  |  | *Helotiales* sp. 1 | 0.000 | 0.000 | 0.000 | 0.765 | 0.173 |
|  |  |  | *Nectriaceae* sp. | 0.000 | 0.000 | 0.086 | 0.537 | 0.144 |
|  |  |  | *Didymosphaeriaceae* sp. | 0.136 | 0.224 | 0.000 | 0.115 | 0.126 |
|  |  |  | *Verrucaria* sp. | 0.000 | 0.000 | 0.000 | 0.551 | 0.124 |
|  |  |  | *Candida parapsilosis* | 0.015 | 0.235 | 0.042 | 0.117 | 0.122 |
|  |  |  | *Trichoderma* sp. | 0.000 | 0.197 | 0.000 | 0.188 | 0.111 |
|  |  |  | *Gliocephalotrichum cylindrosporum* | 0.000 | 0.282 | 0.000 | 0.000 | 0.098 |
|  |  |  | *Cheilymenia theleboloides* | 0.000 | 0.245 | 0.000 | 0.000 | 0.085 |
|  |  |  | *Lipomyces yarrowii* | 0.000 | 0.000 | 0.000 | 0.275 | 0.062 |
|  |  |  | *Didymellaceae* sp. | 0.000 | 0.000 | 0.000 | 0.205 | 0.046 |
|  |  |  | *Aspergillus ellipticus* | 0.000 | 0.114 | 0.000 | 0.000 | 0.040 |
|  |  |  | *Rhinocladiella* sp. | 0.000 | 0.000 | 0.149 | 0.000 | 0.040 |
|  |  |  | *Talaromyces clemensii* | 0.000 | 0.114 | 0.000 | 0.000 | 0.040 |
|  |  |  | *Sporocadaceae* sp. | 0.000 | 0.104 | 0.000 | 0.000 | 0.036 |
|  |  |  | *Clonostachys rosea* | 0.000 | 0.097 | 0.000 | 0.000 | 0.034 |
|  |  |  | *Trichoderma spirale* | 0.000 | 0.000 | 0.000 | 0.147 | 0.033 |
|  |  |  | *Periconia epilithographicola* | 0.000 | 0.000 | 0.000 | 0.143 | 0.032 |
|  |  |  | *Chaetothyriales* sp. 1 | 0.000 | 0.000 | 0.007 | 0.126 | 0.030 |
|  |  |  | *Gorgomyces honrubiae* | 0.000 | 0.000 | 0.000 | 0.131 | 0.030 |
|  |  |  | *Xylonomycetes* sp. | 0.000 | 0.000 | 0.000 | 0.117 | 0.027 |
|  |  |  | *Chaetomiaceae* sp. | 0.000 | 0.000 | 0.000 | 0.116 | 0.026 |
|  |  |  | *Fusarium* sp. | 0.000 | 0.072 | 0.000 | 0.000 | 0.025 |
|  |  |  | *Tolypocladium* sp. | 0.000 | 0.000 | 0.032 | 0.065 | 0.023 |
|  |  |  | *Pyrenochaeta* sp. | 0.000 | 0.065 | 0.000 | 0.000 | 0.023 |
|  |  |  | *Saccharomyces* sp. | 0.096 | 0.000 | 0.000 | 0.022 | 0.020 |
|  |  |  | *Herpotrichiellaceae* sp. 1 | 0.000 | 0.000 | 0.000 | 0.083 | 0.019 |
|  |  |  | *Verrucaria alpicola* | 0.000 | 0.000 | 0.000 | 0.083 | 0.019 |
|  |  |  | *Helotiaceae* sp. | 0.000 | 0.000 | 0.012 | 0.062 | 0.017 |
|  |  |  | *Microdochium phragmitis* | 0.000 | 0.000 | 0.000 | 0.070 | 0.016 |
|  |  |  | *Oidiodendron truncatum* | 0.000 | 0.000 | 0.000 | 0.067 | 0.015 |
|  |  |  | *Protomyces inouyei* | 0.093 | 0.000 | 0.000 | 0.000 | 0.015 |
|  |  |  | *Orbiliaceae* sp. | 0.000 | 0.000 | 0.053 | 0.000 | 0.014 |
|  |  |  | *Orbiliales* sp. 1 | 0.000 | 0.000 | 0.000 | 0.060 | 0.014 |
|  |  |  | *Leohumicola* sp. | 0.000 | 0.000 | 0.000 | 0.060 | 0.014 |
|  |  |  | *Alatospora acuminata* | 0.000 | 0.000 | 0.000 | 0.058 | 0.013 |
|  |  |  | *Sordariomycetes* sp. | 0.000 | 0.000 | 0.000 | 0.050 | 0.011 |
|  |  |  | *Sarocladium* sp. | 0.000 | 0.000 | 0.000 | 0.042 | 0.010 |
|  |  |  | *Sporormiaceae* sp. | 0.000 | 0.000 | 0.000 | 0.041 | 0.009 |
|  |  |  | *Cyberlindnera jadinii* | 0.000 | 0.000 | 0.000 | 0.040 | 0.009 |
|  |  |  | *Toxicocladosporium* sp. | 0.000 | 0.000 | 0.034 | 0.000 | 0.009 |
|  |  |  | *Aspergillus thermomutatus* | 0.000 | 0.000 | 0.000 | 0.039 | 0.009 |
|  |  |  | *Leotiomycetes* sp. 1 | 0.000 | 0.000 | 0.000 | 0.039 | 0.009 |
|  |  |  | *Penicillium coffeae* | 0.053 | 0.000 | 0.000 | 0.000 | 0.009 |
|  |  |  | *Penicillium mallochii* | 0.000 | 0.000 | 0.000 | 0.037 | 0.008 |
|  |  |  | *Porpidia* sp. | 0.000 | 0.000 | 0.000 | 0.036 | 0.008 |
|  |  |  | *Stagonospora trichophoricola* | 0.000 | 0.000 | 0.000 | 0.035 | 0.008 |
|  |  |  | *Lipomyces kononenkoae* | 0.000 | 0.000 | 0.000 | 0.035 | 0.008 |
|  |  |  | *Pyricularia oryzae* | 0.019 | 0.000 | 0.016 | 0.000 | 0.007 |
|  |  |  | *Beauveria* sp. | 0.000 | 0.000 | 0.000 | 0.032 | 0.007 |
|  |  |  | *Bionectriaceae* sp. | 0.000 | 0.000 | 0.000 | 0.032 | 0.007 |
|  |  |  | *Neonectria candida* | 0.000 | 0.000 | 0.000 | 0.032 | 0.007 |
|  |  |  | *Cladophialophora* sp. | 0.000 | 0.000 | 0.000 | 0.029 | 0.007 |
|  |  |  | *Lipomyces starkeyi* | 0.000 | 0.000 | 0.000 | 0.028 | 0.006 |
|  |  |  | *Archaeorhizomyces* sp. | 0.000 | 0.000 | 0.000 | 0.027 | 0.006 |
|  |  |  | *Leptosphaeria sclerotioides* | 0.000 | 0.000 | 0.000 | 0.027 | 0.006 |
|  |  |  | *Cladophialophora minutissima* | 0.000 | 0.000 | 0.000 | 0.026 | 0.006 |
|  |  |  | *Dothideales* sp. | 0.000 | 0.000 | 0.000 | 0.025 | 0.006 |
|  |  |  | *Coniosporium* sp. | 0.000 | 0.000 | 0.000 | 0.024 | 0.005 |
|  |  |  | *Coniochaeta* sp. | 0.000 | 0.000 | 0.000 | 0.023 | 0.005 |
|  |  |  | *Saccharomycopsis fibuligera* | 0.000 | 0.000 | 0.000 | 0.022 | 0.005 |
|  |  |  | *Helotiales* sp. 2 | 0.000 | 0.000 | 0.000 | 0.020 | 0.004 |
|  |  |  | *Clavicipitaceae* sp. 1 | 0.000 | 0.000 | 0.000 | 0.019 | 0.004 |
|  |  |  | *Herpotrichiellaceae* sp. 2 | 0.000 | 0.000 | 0.000 | 0.018 | 0.004 |
|  |  |  | *Annulatascaceae* sp. | 0.000 | 0.000 | 0.000 | 0.017 | 0.004 |
|  |  |  | *Capnodiales* sp. | 0.000 | 0.000 | 0.000 | 0.017 | 0.004 |
|  |  |  | *Pseudeurotiaceae* sp. | 0.000 | 0.000 | 0.000 | 0.017 | 0.004 |
|  |  |  | *Diatrypaceae* sp. | 0.023 | 0.000 | 0.000 | 0.000 | 0.004 |
|  |  |  | *Candida* sp. | 0.000 | 0.000 | 0.000 | 0.016 | 0.004 |
|  |  |  | *Buellia russa* | 0.022 | 0.000 | 0.000 | 0.000 | 0.004 |
|  |  |  | *Hyaloscypha* sp. | 0.000 | 0.000 | 0.000 | 0.015 | 0.003 |
|  |  |  | *Debaryomyces* sp. | 0.000 | 0.000 | 0.000 | 0.013 | 0.003 |
|  |  |  | *Gyoerffyella entomobryoides* | 0.000 | 0.000 | 0.000 | 0.013 | 0.003 |
|  |  |  | *Pleosporaceae* sp. | 0.000 | 0.000 | 0.000 | 0.013 | 0.003 |
|  |  |  | *Hypocreales* sp. | 0.000 | 0.000 | 0.000 | 0.012 | 0.003 |
|  |  |  | *Periconia byssoides* | 0.000 | 0.000 | 0.000 | 0.012 | 0.003 |
|  |  |  | *Pezicula* sp. | 0.000 | 0.000 | 0.000 | 0.011 | 0.003 |
|  |  |  | *Chloridium* sp. | 0.000 | 0.000 | 0.000 | 0.010 | 0.002 |
|  |  |  | *Cenococcum geophilum* | 0.000 | 0.000 | 0.000 | 0.010 | 0.002 |
|  |  |  | *Wickerhamomyces anomalus* | 0.000 | 0.000 | 0.000 | 0.009 | 0.002 |
|  |  |  | *Iodophanus carneus* | 0.000 | 0.000 | 0.000 | 0.009 | 0.002 |
|  |  |  | *Phialocephala* sp. | 0.000 | 0.000 | 0.000 | 0.009 | 0.002 |
|  |  |  | *Clavicipitaceae* sp. 2 | 0.000 | 0.000 | 0.000 | 0.007 | 0.002 |
|  |  |  | *Dothideomycetes* sp. 1 | 0.000 | 0.000 | 0.000 | 0.005 | 0.001 |
|  |  |  | *Galactomyces reessii* | 0.000 | 0.000 | 0.000 | 0.002 | 0.000 |
|  |  | *Basidiomycota* | *Camptobasidiaceae* sp. | 22.120 | 0.000 | 0.000 | 0.918 | 3.777 |
|  |  |  | *Leucosporidiales* sp. 1 | 13.957 | 0.000 | 0.000 | 0.000 | 2.252 |
|  |  |  | *Phenoliferia psychrophila* | 13.445 | 0.000 | 0.000 | 0.000 | 2.170 |
|  |  |  | *Glaciozyma* sp. | 3.019 | 0.000 | 0.000 | 0.068 | 0.503 |
|  |  |  | *Glaciozyma martinii* | 2.537 | 0.000 | 0.000 | 0.050 | 0.421 |
|  |  |  | *Glaciozyma antarctica* | 0.655 | 0.000 | 0.064 | 0.751 | 0.292 |
|  |  |  | *Goffeauzyma gastrica* | 0.000 | 0.763 | 0.000 | 0.000 | 0.265 |
|  |  |  | *Mrakia* sp. | 0.768 | 0.000 | 0.000 | 0.409 | 0.216 |
|  |  |  | *Tremellomycetes* sp. 1 | 1.128 | 0.000 | 0.000 | 0.000 | 0.182 |
|  |  |  | *Mrakia psychrophila* | 0.713 | 0.000 | 0.000 | 0.167 | 0.153 |
|  |  |  | *Mrakia niccombsii* | 0.677 | 0.000 | 0.000 | 0.000 | 0.109 |
|  |  |  | *Cutaneotrichosporon debeurmannianum* | 0.004 | 0.235 | 0.000 | 0.029 | 0.089 |
|  |  |  | *Malassezia restricta* | 0.086 | 0.000 | 0.008 | 0.313 | 0.087 |
|  |  |  | *Leucosporidiales* sp. 2 | 0.496 | 0.000 | 0.000 | 0.000 | 0.080 |
|  |  |  | *Dioszegia fristingensis* | 0.483 | 0.000 | 0.000 | 0.000 | 0.078 |
|  |  |  | *Microbotryomycetes* sp. | 0.000 | 0.000 | 0.015 | 0.315 | 0.075 |
|  |  |  | *Leucosporidium creatinivorum* | 0.000 | 0.000 | 0.000 | 0.281 | 0.063 |
|  |  |  | *Vishniacozyma victoriae* | 0.351 | 0.000 | 0.000 | 0.000 | 0.057 |
|  |  |  | *Holocotylon* sp. | 0.349 | 0.000 | 0.000 | 0.000 | 0.056 |
|  |  |  | *Glaciozyma watsonii* | 0.000 | 0.000 | 0.000 | 0.243 | 0.055 |
|  |  |  | *Solicoccozyma terricola* | 0.148 | 0.000 | 0.000 | 0.097 | 0.046 |
|  |  |  | *Holtermanniella takashimae* | 0.266 | 0.000 | 0.000 | 0.000 | 0.043 |
|  |  |  | *Tremellomycetes* sp. 2 | 0.235 | 0.000 | 0.000 | 0.000 | 0.038 |
|  |  |  | *Psathyrellaceae* sp. | 0.000 | 0.000 | 0.000 | 0.112 | 0.025 |
|  |  |  | *Wallemia tropicalis* | 0.000 | 0.000 | 0.000 | 0.087 | 0.020 |
|  |  |  | *Phanerochaete citri* | 0.073 | 0.000 | 0.000 | 0.034 | 0.019 |
|  |  |  | *Malassezia globosa* | 0.000 | 0.000 | 0.000 | 0.068 | 0.015 |
|  |  |  | *Agaricomycetes* sp. 1 | 0.000 | 0.000 | 0.000 | 0.067 | 0.015 |
|  |  |  | *Coprinopsis* sp. | 0.000 | 0.000 | 0.049 | 0.000 | 0.013 |
|  |  |  | *Porostereum spadiceum* | 0.000 | 0.000 | 0.000 | 0.052 | 0.012 |
|  |  |  | *Dioszegia* sp. | 0.062 | 0.000 | 0.000 | 0.000 | 0.010 |
|  |  |  | *Malassezia arunalokei* | 0.000 | 0.000 | 0.000 | 0.040 | 0.009 |
|  |  |  | *Naganishia friedmannii* | 0.000 | 0.000 | 0.000 | 0.029 | 0.007 |
|  |  |  | *Malassezia sympodialis* | 0.023 | 0.000 | 0.000 | 0.012 | 0.006 |
|  |  |  | *Genolevuria amylolytica* | 0.000 | 0.000 | 0.000 | 0.023 | 0.005 |
|  |  |  | *Bullera penniseticola* | 0.000 | 0.000 | 0.000 | 0.022 | 0.005 |
|  |  |  | *Clavaria* sp. | 0.000 | 0.000 | 0.000 | 0.016 | 0.004 |
|  |  |  | *Renatobasidium* sp. | 0.000 | 0.000 | 0.000 | 0.014 | 0.003 |
|  |  |  | *Acaromyces ingoldii* | 0.000 | 0.000 | 0.000 | 0.013 | 0.003 |
|  |  |  | *Rhodotorula pacifica* | 0.000 | 0.000 | 0.010 | 0.000 | 0.003 |
|  |  |  | *Tremellales* sp. | 0.013 | 0.000 | 0.000 | 0.000 | 0.002 |
|  |  |  | *Trametes hirsuta* | 0.000 | 0.000 | 0.000 | 0.009 | 0.002 |
|  |  |  | *Filobasidiales* sp. | 0.009 | 0.000 | 0.000 | 0.000 | 0.001 |
|  |  |  | *Piskurozyma* sp. | 0.005 | 0.000 | 0.000 | 0.000 | 0.001 |
|  |  | *Mortierellomycota* | *Mortierellales* sp. 1 | 0.000 | 0.367 | 0.000 | 0.000 | 0.127 |
|  |  |  | *Mortierella antarctica* | 0.000 | 0.000 | 0.000 | 0.503 | 0.114 |
|  |  |  | *Mortierella* sp. 1 | 0.000 | 0.000 | 0.000 | 0.489 | 0.110 |
|  |  |  | *Mortierella minutissima* | 0.000 | 0.000 | 0.000 | 0.335 | 0.076 |
|  |  |  | *Mortierella* sp. 2 | 0.000 | 0.000 | 0.000 | 0.156 | 0.035 |
|  |  |  | *Mortierella horticola* | 0.000 | 0.000 | 0.000 | 0.088 | 0.020 |
|  |  |  | *Mortierella pseudozygospora* | 0.000 | 0.000 | 0.000 | 0.050 | 0.011 |
|  |  |  | *Mortierella humilis* | 0.000 | 0.000 | 0.000 | 0.050 | 0.011 |
|  |  |  | *Mortierella basiparvispora* | 0.000 | 0.000 | 0.000 | 0.036 | 0.008 |
|  |  |  | *Mortierella alpina* | 0.000 | 0.000 | 0.000 | 0.029 | 0.007 |
|  |  |  | *Mortierella gemmifera* | 0.000 | 0.000 | 0.000 | 0.018 | 0.004 |
|  |  | *Chytridiomycota* | *Betamyces* sp. | 0.373 | 1.446 | 1.464 | 2.444 | 1.503 |
|  |  |  | *Spizellomycetales* sp. 1 | 0.000 | 0.000 | 4.221 | 0.000 | 1.119 |
|  |  |  | *Lobulomycetales* sp. | 0.000 | 0.000 | 1.763 | 0.798 | 0.648 |
|  |  |  | *Chytridium* sp. | 0.000 | 0.715 | 0.000 | 0.099 | 0.271 |
|  |  |  | *Rhizophydiales* sp. 1 | 0.354 | 0.000 | 0.172 | 0.215 | 0.151 |
|  |  |  | *Lobulomycetes* sp. | 0.000 | 0.000 | 0.342 | 0.035 | 0.098 |
|  |  |  | *Rhizophydiales* sp. 2 | 0.036 | 0.240 | 0.007 | 0.000 | 0.091 |
|  |  |  | *Chytridiales* sp. 1 | 0.000 | 0.000 | 0.028 | 0.000 | 0.007 |
|  |  | *Monoblepharomycota* | *Monoblepharidales* sp. | 3.566 | 0.000 | 0.000 | 0.097 | 0.597 |
|  |  |  | *Sanchytrium* sp. | 0.000 | 0.000 | 0.081 | 1.112 | 0.273 |
|  |  |  | *Sanchytriaceae* sp. | 0.000 | 0.000 | 0.000 | 0.051 | 0.011 |
|  |  |  | *Monoblepharis* sp. | 0.000 | 0.000 | 0.000 | 0.027 | 0.006 |
|  |  | *Rozellomycota* | *Rozellomycotina* sp. 1 | 0.000 | 0.275 | 1.653 | 0.209 | 0.581 |
|  |  |  | *Rozellomycotina* sp. 2 | 0.000 | 0.000 | 0.000 | 0.625 | 0.141 |
|  |  |  | *Rozellomycotina* sp. 3 | 0.000 | 0.000 | 0.186 | 0.000 | 0.049 |
|  |  |  | *Rozellomycotina* sp. 4 | 0.000 | 0.000 | 0.000 | 0.117 | 0.027 |
|  |  | *Mucoromycota* | *Pirella circinans* | 0.000 | 0.000 | 0.000 | 0.012 | 0.003 |
|  |  |  | *Endogonomycetes* sp. | 0.000 | 0.000 | 0.000 | 0.004 | 0.001 |
|  |  | *Zoopagomycota* | *Zoopagales* sp. | 0.000 | 0.000 | 0.000 | 0.004 | 0.001 |
|  |  |  | *Acaulopage* sp. | 0.000 | 0.000 | 0.000 | 0.003 | 0.001 |
|  |  | *Aphelidiomycota* | *Aphelidiomycetes* sp. | 0.000 | 0.000 | 0.458 | 0.420 | 0.216 |
|  |  | *Blastocladiomycota* | *Paraphysoderma* sp. | 0.000 | 0.000 | 0.596 | 0.000 | 0.158 |
|  |  | *Basidiobolomycota* | *Basidiobolales* sp. | 0.000 | 0.000 | 0.021 | 0.074 | 0.022 |
|  |  | *Glomeromycota* | *Scutellospora* sp. | 0.000 | 0.000 | 0.000 | 0.006 | 0.001 |
|  | Straminopila | *Oomycota* | *Saprolegniaceae* sp. | 0.000 | 0.000 | 0.000 | 0.020 | 0.004 |
| BLAST | Fungi | Unknown | Fungal sp. 1 | 20.205 | 1.136 | 66.391 | 15.467 | 24.757 |
|  |  |  | Fungal sp. 2 | 2.614 | 3.862 | 3.118 | 8.282 | 4.462 |
|  |  | *Ascomycota* | *Saccharomycetales* sp. 1 | 0.000 | 0.000 | 0.000 | 3.842 | 0.868 |
|  |  |  | *Sordariales* sp. | 0.000 | 0.000 | 1.469 | 0.027 | 0.396 |
|  |  |  | *Ascomycota* sp.1 | 0.000 | 0.000 | 0.000 | 0.860 | 0.194 |
|  |  |  | *Eurotiales* sp. | 0.000 | 0.000 | 0.000 | 0.243 | 0.055 |
|  |  |  | *Ascomycota* sp. 2 | 0.000 | 0.000 | 0.000 | 0.102 | 0.023 |
|  |  |  | *Neolectales* sp. | 0.000 | 0.000 | 0.000 | 0.091 | 0.021 |
|  |  |  | *Saccharomycetales* sp. 2 | 0.000 | 0.000 | 0.000 | 0.043 | 0.010 |
|  |  |  | *Orbiliales* sp. 2 | 0.036 | 0.000 | 0.000 | 0.000 | 0.006 |
|  |  |  | *Letiomyceta* sp. | 0.000 | 0.000 | 0.000 | 0.020 | 0.004 |
|  |  |  | *Chaetothyriales* sp. 2 | 0.000 | 0.000 | 0.016 | 0.000 | 0.004 |
|  |  |  | *Leotiomycetes* sp. 2 | 0.000 | 0.000 | 0.000 | 0.018 | 0.004 |
|  |  |  | *Dothideomycetes* sp. 2 | 0.000 | 0.000 | 0.011 | 0.003 | 0.004 |
|  |  |  | *Leptodiscella* sp. | 0.000 | 0.000 | 0.007 | 0.000 | 0.002 |
|  |  | *Basidiomycota* | *Agaricales* sp. | 0.000 | 0.000 | 6.461 | 0.200 | 1.759 |
|  |  |  | *Polyporales* sp. | 0.000 | 0.000 | 0.000 | 0.451 | 0.102 |
|  |  |  | *Basidiomycota* sp. | 0.000 | 0.000 | 0.095 | 0.062 | 0.039 |
|  |  |  | *Boletales* sp. | 0.000 | 0.000 | 0.000 | 0.058 | 0.013 |
|  |  |  | *Gomphales* sp. | 0.000 | 0.000 | 0.025 | 0.000 | 0.007 |
|  |  |  | *Trechisporales* sp. | 0.000 | 0.000 | 0.000 | 0.017 | 0.004 |
|  |  |  | *Agaricomycetes* sp. 2 | 0.000 | 0.000 | 0.000 | 0.008 | 0.002 |
|  |  | *Chytridiomycota* | *Chytridiales* sp. 2 | 0.000 | 0.000 | 0.000 | 3.982 | 0.900 |
|  |  |  | *Rhizophydiales* sp. 3 | 0.000 | 0.000 | 0.138 | 1.964 | 0.481 |
|  |  |  | *Chytridiomycota* sp. | 0.000 | 0.663 | 0.061 | 0.176 | 0.286 |
|  |  |  | *Rhizophydiales* sp. 4 | 0.000 | 0.000 | 0.272 | 0.000 | 0.072 |
|  |  |  | *Spizellomycetales* sp. 2 | 0.000 | 0.000 | 0.027 | 0.192 | 0.051 |
|  |  | *Mucoromycota* | *Mortierellales* sp. 2 | 0.000 | 0.000 | 0.847 | 0.261 | 0.284 |
|  |  |  | *Cryptomycota* sp. | 0.000 | 0.000 | 0.403 | 0.021 | 0.112 |
|  |  |  | *Glomerales* sp. | 0.000 | 0.000 | 0.000 | 0.041 | 0.009 |
|  |  |  | *Archaeosporales* sp. | 0.056 | 0.000 | 0.000 | 0.000 | 0.009 |
|  |  |  | *Glomeromycotina* sp. | 0.000 | 0.000 | 0.000 | 0.011 | 0.003 |
|  |  | *Blastocladiomycota* | *Blastocladiales* sp. | 0.000 | 0.000 | 0.000 | 0.260 | 0.059 |
|  |  | *Zoopagomycota* | *Entomophthoromycota* sp. | 0.007 | 0.000 | 0.000 | 0.027 | 0.007 |
|  | Stramenopila | *Bacillariophyta* | *Bacillariophyta* sp. 1 | 0.000 | 0.000 | 0.000 | 1.063 | 0.240 |
|  |  |  | *Bacillariophyceae* sp. | 0.000 | 0.000 | 0.000 | 0.056 | 0.013 |
|  |  |  | *Bacillariophyta* sp. 2 | 0.000 | 0.007 | 0.000 | 0.000 | 0.002 |
| **Total** |  |  |  | **100** | **100** | **100** | **100** | **100** |

Green indicates dominant, blue intermediate, and orange minor relative abundance (see Methods).
