# Supplementary material for: Diversity, distribution and ecology of fungal communities present in Antarctic lake sediments uncovered by DNA metabarcoding
Source: Sci Rep. 2022 May 19;12:8407. doi: 10.1038/s41598-022-12290-6 (PMC9120451; doi:10.1038/s41598-022-12290-6)
Supplement: Supplementary file 7 — Supplementary Information 7. [file 41598_2022_12290_MOESM7_ESM.docx]

**Diversity, distribution and ecology of fungal communities present in Antarctic lake sediments uncovered by DNA metabarcoding**

Láuren Machado Drumond de Souza, Juan Manuel Lirio, Silvia H. Coria, Fabyano Alvares Cardoso Lopes, Peter Convey, Micheline Carvalho-Silva, Fábio Soares de Oliveira, Carlos Augusto Rosa, Paulo EAS Câmara and Luiz Henrique Rosa

**Supplementary Table S3.** Ecological profiles obtained from the FUNGuild database and specific citations at generic level of the fungi detected in sediment samples obtained from Skua Lake (Elephant Island), Soto Lake (Deception Island), Katerina Lake and Florencia Lake (James Ross Island).

| **Genus** | **Skua Lake, Elephant Island** | **Soto Lake, Deception Island** | **Katerina Lake, James Ross Island** | **Florencia Lake, James Ross Island** | **Total** | **Trophic mode** | **Guild*** |
| --- | --- | --- | --- | --- | --- | --- | --- |
| *Acaromyces* | 0.000 | 0.000 | 0.000 | 0.013 | 0.003 | Symbiotic- Pathogenic | Endophyte-Acaropathogenic^48,49^ |
| *Acaulopage* | 0.000 | 0.000 | 0.000 | 0.003 | 0.001 | Pathogenic | Animal Pathogen^35^ |
| *Acremonium* | 0.000 | 0.000 | 0.000 | 4.928 | 1.113 | Pathogenic-Saprotrophic-Symbiotic | Animal Pathogen- Endophyte-Fungal^35^ Parasite-Plant Pathogen-Wood^35^ Saprotrophic^35^ |
| *Alatospora* | 0.000 | 0.000 | 0.000 | 0.058 | 0.013 | Saprotrophic | Undefined Saprotrophic^35^ |
| *Antarctomyces* | 0.730 | 0.000 | 0.194 | 0.080 | 0.187 | Saprotrophic | Undefined Saprotrophic^35^ |
| *Archaeorhizomyces* | 0.000 | 0.000 | 0.000 | 0.027 | 0.006 | Saprotrophic | Soil Saprotrophic^50^ |
| *Aspergillus* | 0.000 | 0.057 | 0.000 | 0.019 | 0.024 | Pathogenic-Saprotrophic | Animal Pathogen-Undefined Saprotrophic^35^ |
| *Beauveria* | 0.000 | 0.000 | 0.000 | 0.032 | 0.007 | Pathogenic | Animal Pathogen^35^ |
| *Betamyces* | 0.373 | 1.446 | 1.464 | 2.444 | 1.503 | Saprotrophic | Freshwater and Soil Saprotrophic^51^ |
| *Buellia* | 0.022 | 0.000 | 0.000 | 0.000 | 0.004 | Symbiotic | Lichenized^35^ |
| *Bullera* | 0.000 | 0.000 | 0.000 | 0.022 | 0.005 | Symbiotic | Endophyte^52^ |
| *Candida* | 0.007 | 0.117 | 0.021 | 0.066 | 0.063 | Pathogenic | Animal Pathogen^35^ |
| *Cenococcum* | 0.000 | 0.000 | 0.000 | 0.010 | 0.002 | Symbiotic | Ectomycorrhizal^35^ |
| *Cheilymenia* | 0.000 | 0.245 | 0.000 | 0.000 | 0.085 | Saprotrophic | Dung Saprotrophic-Undefined Saprotrophic^35^ |
| *Chloridium* | 0.000 | 0.000 | 0.000 | 0.010 | 0.002 | Pathogenic-Saprotrophic-Symbiotic | Ectomycorrhizal-Endophyte-Plant Pathogen-Wood Saprotrophic^35^ |
| *Chytridium* | 0.000 | 0.715 | 0.000 | 0.099 | 0.271 | Pathogenic | Plant Pathogen^35^ |
| *Ciliophora* | 1.008 | 0.000 | 0.043 | 1.854 | 0.593 | Symbiotic | Endophyte^53^ |
| *Cladophialophora* | 0.000 | 0.000 | 0.000 | 0.028 | 0.006 | Saprotrophic | Undefined Saprotrophic^35^ |
| *Cladosporium* | 0.000 | 0.343 | 0.000 | 0.850 | 0.311 | Symbiotic | Endophyte^35^ |
| *Clathrosphaerina* | 0.000 | 0.390 | 0.000 | 4.478 | 1.148 | Saprotrophic | Undefined Saprotrophic^35^ |
| *Clavaria* | 0.000 | 0.000 | 0.000 | 0.016 | 0.004 | Saprotrophic | Undefined Saprotrophic^35^ |
| *Clonostachys* | 0.000 | 0.097 | 0.000 | 0.000 | 0.034 | Pathogenic | Plant Pathogen^35^ |
| *Coniochaeta* | 0.000 | 0.000 | 0.000 | 0.023 | 0.005 | Pathogenic-Saprotrophic-Symbiotic | Animal Pathogen-Dung Saprotrophic-Endophyte-Lichen Parasite-Plant Pathogen-Undefined Saprotrophic^35^ |
| *Coniosporium* | 0.000 | 0.000 | 0.000 | 0.024 | 0.005 | Saprotrophic | Undefined Saprotrophic^54^ |
| *Coprinopsis* | 0.000 | 0.000 | 0.049 | 0.000 | 0.013 | Saprotrophic | Undefined Saprotrophic^55^ |
| *Cutaneotrichosporon* | 0.004 | 0.235 | 0.000 | 0.029 | 0.089 | Saprotrophic | Undefined Saprotrophic^56^ |
| *Cyberlindnera* | 0.000 | 0.000 | 0.000 | 0.040 | 0.009 | Symbiotic | Insect Symbiont^57^ |
| *Dactylonectria* | 0.000 | 39.311 | 0.000 | 0.017 | 13.663 | Saprotrophic-Pathogenic | Soil Saprotrophic- Plant Pathogen^58^ |
| *Debaryomyces* | 0.000 | 0.000 | 0.000 | 0.013 | 0.003 | Saprotrophic | Undefined Saprotrophic^35^ |
| *Dioszegia* | 0.272 | 0.000 | 0.000 | 0.000 | 0.044 | Saprotrophic | Undefined Saprotrophic^35^ |
| *Fusarium* | 0.000 | 2.499 | 0.000 | 0.037 | 0.876 | Pathogenic-Saprotrophic-Symbiotic | Animal Pathogen-Endophyte-Lichen^35^ Parasite-Plant Pathogen-Soil^35^ Saprotrophic-Wood Saprotrophic^35^ |
| *Galactomyces* | 0.000 | 0.000 | 0.000 | 0.002 | 0.000 | Pathogenic | Plant Pathogen^35^ |
| *Genolevuria* | 0.000 | 0.000 | 0.000 | 0.023 | 0.005 | Saprotrophic | Undefined Saprotrophic^59^ |
| *Glaciozyma* | 1.553 | 0.000 | 0.016 | 0.278 | 0.318 | Saprotrophic | Undefined Saprotrophic^60^ |
| *Gliocephalotrichum* | 0.000 | 0.282 | 0.000 | 0.000 | 0.098 | Saprotrophic | Undefined Saprotrophic^35^ |
| *Goffeauzyma* | 0.000 | 0.763 | 0.000 | 0.000 | 0.265 | Saprotrophic | Soil-Acid rock drainage-Acidic water^61^ |
| *Gorgomyces* | 0.000 | 0.000 | 0.000 | 0.131 | 0.030 | Pathogenic | Nematophagous^48^ |
| *Gyoerffyella* | 0.000 | 0.000 | 0.000 | 0.013 | 0.003 | Saprotrophic | Undefined Saprotrophic^35^ |
| *Holocotylon* | 0.349 | 0.000 | 0.000 | 0.000 | 0.056 | Saprotrophic | Undefined Saprotrophic^35^ |
| *Holtermanniella* | 0.266 | 0.000 | 0.000 | 0.000 | 0.043 | Symbiotic | Plant Symbiotic^62^ |
| *Hyaloscypha* | 0.000 | 0.000 | 0.000 | 0.015 | 0.003 | Saprotrophic | Undefined Saprotrophic^35^ |
| *Iodophanus* | 0.000 | 0.000 | 0.000 | 0.009 | 0.002 | Saprotrophic | Undefined Saprotrophic^35^ |
| *Leohumicola* | 0.000 | 0.000 | 0.000 | 0.060 | 0.014 | Saprotrophic | Undefined Saprotrophic^35^ |
| *Leptodiscella* | 0.000 | 0.000 | 0.007 | 0.000 | 0.002 | Saprotrophic | Undefined Saprotrophic^35^ |
| *Leptosphaeria* | 0.000 | 0.000 | 0.000 | 0.027 | 0.006 | Pathogenic | Plant Pathogen^35^ |
| *Leucosporidium* | 0.000 | 0.000 | 0.000 | 0.281 | 0.063 | Saprotrophic | Soil Saprotrophic-Undefined Saprotrophic^35^ |
| *Lipomyces* | 0.000 | 0.000 | 0.000 | 0.113 | 0.025 | Saprotrophic | Soil Saprotrophic-Insect frass^63^ |
| *Malassezia* | 0.027 | 0.000 | 0.002 | 0.108 | 0.029 | Pathogenic | Animal Pathogen^35^ |
| *Microdochium* | 0.000 | 0.000 | 0.000 | 0.070 | 0.016 | Pathogenic-Symbiotic | Endophyte-Plant Pathogen^35^ |
| *Monoblepharis* | 0.000 | 0.000 | 0.000 | 0.027 | 0.006 | Saprotrophic | Undefined Saprotrophic^35^ |
| *Mortierella* | 0.000 | 0.000 | 0.000 | 0.175 | 0.040 | Saprotrophic-Symbiotic | Endophyte-Litter Saprotrophic-Soil^35^ Saprotrophic-Undefined Saprotrophic^35^ |
| *Mrakia* | 0.719 | 0.000 | 0.000 | 0.192 | 0.159 | Saprotrophic | Soil Saprotrophic-Undefined Saprotrophic^35^ |
| *Naganishia* | 0.000 | 0.000 | 0.000 | 0.029 | 0.007 | Saprotrophic | Soil-Flowers^64,65^ |
| *Neoascochyta* | 1.513 | 0.406 | 0.137 | 0.542 | 0.544 | Pathogenic | Plant Pathogen^66^ |
| *Neonectria* | 0.000 | 0.000 | 0.000 | 0.032 | 0.007 | Pathogenic | Plant Pathogen^35^ |
| *Oidiodendron* | 0.000 | 0.000 | 0.000 | 0.067 | 0.015 | Pathogenic-Symbiotic | Ericoid Mycorrhizal^35^ |
| *Paraphysoderma* | 0.000 | 0.000 | 0.596 | 0.000 | 0.158 | Pathogenic | Plant Pathogen^35^ |
| *Penicillium* | 0.665 | 0.132 | 0.135 | 0.233 | 0.242 | Saprotrophic | Dung Saprotrophic-Undefined^35^ Saprotrophic-Wood Saprotrophic^35^ |
| *Periconia* | 0.000 | 0.000 | 0.000 | 0.077 | 0.017 | Pathogenic-Saprotrophic-Symbiotic | Endophyte-Plant Pathogen-Wood Saprotrophic^35^ |
| *Pezicula* | 0.000 | 0.000 | 0.000 | 0.011 | 0.003 | Saprotrophic-Symbiotic-Pathogenic | Wood Saprotrophic-Endophyte-Plant Pathogen^67^ |
| *Phanerochaete* | 0.073 | 0.000 | 0.000 | 0.034 | 0.019 | Saprotrophic | Wood Saprotrophic^35^ |
| *Phenoliferia* | 13.445 | 0.000 | 0.000 | 0.000 | 2.170 | Symbiotic-Saprotrophic | Plant-Cold habitats^68^ |
| *Phialocephala* | 0.000 | 0.000 | 0.000 | 0.009 | 0.002 | Symbiotic | Endophyte^35^ |
| *Pirella* | 0.000 | 0.000 | 0.000 | 0.012 | 0.003 | Saprotrophic | Undefined Saprotrophic^35^ |
| *Piskurozyma* | 0.005 | 0.000 | 0.000 | 0.000 | 0.001 | Saprotrophic | Soil Saprotrophic^69^ |
| *Porostereum* | 0.000 | 0.000 | 0.000 | 0.052 | 0.012 | Saprotrophic | Undefined Saprotrophic^35^ |
| *Porpidia* | 0.000 | 0.000 | 0.000 | 0.036 | 0.008 | Symbiotic | Lichenized^35^ |
| *Protomyces* | 0.093 | 0.000 | 0.000 | 0.000 | 0.015 | Pathogenic | Plant Pathogen^35^ |
| *Pseudeurotium* | 1.130 | 0.000 | 0.000 | 11.814 | 2.852 | Saprotrophic | Undefined Saprotrophic^35^ |
| *Pseudogymnoascus* | 0.694 | 0.207 | 0.049 | 8.302 | 2.073 | Pathogenic-Saprotrophic-Symbiotic | Animal pathogen-Soil Saprotrophic^35^ |
| *Pyrenochaeta* | 0.000 | 0.065 | 0.000 | 0.000 | 0.023 | Pathogenic-Symbiotic-Saprotrophic | Plant Pathogen- Endophyte -Soil^35^ Saprotrophic^70^ |
| *Pyricularia* | 0.019 | 0.000 | 0.016 | 0.000 | 0.007 | Pathogenic | Plant Pathogen^35^ |
| *Renatobasidium* | 0.000 | 0.000 | 0.000 | 0.014 | 0.003 | Saprotrophic | Undefined Saprotrophic^35^ |
| *Rhinocladiella* | 0.000 | 0.000 | 0.149 | 0.000 | 0.040 | Pathogenic-Saprotrophic | Plant Pathogen- Wood Saprotrophic^48^ |
| *Rhodotorula* | 0.000 | 0.000 | 0.010 | 0.000 | 0.003 | Pathogenic-Saprotrophic | Animal Endosymbiont-Animal Pathogen-Endophyte-Plant Pathogen-Undefined Saprotroph^35^ |
| *Saccharomyces* | 0.096 | 0.000 | 0.000 | 0.022 | 0.020 | Saprotrophic | Undefined Saprotrophic^35^ |
| *Saccharomycopsis* | 0.000 | 0.000 | 0.000 | 0.022 | 0.005 | Saprotrophic | Undefined Saprotrophic^71^ |
| *Sanchytrium* | 0.000 | 0.000 | 0.081 | 1.112 | 0.273 | Pathogenic | Algae Pathogen^72^ |
| *Sarocladium* | 0.000 | 0.000 | 0.000 | 0.042 | 0.010 | Saprotrophic | Undefined Saprotrophic^35^ |
| *Scutellinia* | 0.000 | 0.000 | 0.000 | 8.380 | 1.894 | Saprotrophic | Undefined Saprotrophic^35^ |
| *Scutellospora* | 0.000 | 0.000 | 0.000 | 0.006 | 0.001 | Symbiotic | Arbuscular Mycorrhizal^35^ |
| *Solicoccozyma* | 0.148 | 0.000 | 0.000 | 0.097 | 0.046 | Saprotrophic | Soil Saprotrophic^61^ |
| *Stagonospora* | 0.000 | 0.000 | 0.000 | 0.035 | 0.008 | Pathogenic | Plant Pathogen^35^ |
| *Talaromyces* | 0.000 | 20.537 | 2.867 | 0.000 | 7.897 | Saprotrophic | Undefined Saprotrophic^35^ |
| *Tetracladium* | 0.000 | 0.000 | 1.873 | 0.358 | 0.578 | Saprotrophic | Undefined Saprotrophic^35^ |
| *Thelebolus* | 0.000 | 0.000 | 0.000 | 1.437 | 0.325 | Saprotrophic-Symbiotic | Dung Saprotrophic-Endophyte-Undefined Saprotrophic^35^ |
| *Tolypocladium* | 0.000 | 0.000 | 0.032 | 0.065 | 0.023 | Pathogenic-Symbiotic | Animal Pathogen-Clavicipitaceous Endophyte-Fungal Parasite^35^ |
| *Toxicocladosporium* | 0.000 | 0.000 | 0.034 | 0.000 | 0.009 | Pathogenic-Symbiotic | Endophyte-Plant Pathogen^35^ |
| *Trametes* | 0.000 | 0.000 | 0.000 | 0.009 | 0.002 | Saprotrophic | Wood Saprotrophic^35^ |
| *Trichoderma* | 0.000 | 0.099 | 0.000 | 0.167 | 0.072 | Pathogenic-Saprotrophic-Symbiotic | Animal Pathogen-Endophyte-Epiphyte-Fungal Parasite-Plant Pathogen-Wood Saprotrophic^35^ |
| *Verrucaria* | 0.000 | 0.000 | 0.000 | 0.317 | 0.072 | Symbiotic | Lichenized^73^ |
| *Vishniacozyma* | 0.351 | 0.000 | 0.000 | 0.000 | 0.057 | Pathogenic-Saprotrophic-Symbiotic | Animal Pathogen-Endophyte-Epiphyte-Undefined Saprotrophic^35^ |
| *Wallemia* | 0.000 | 0.000 | 0.000 | 0.087 | 0.020 | Saprotrophic | Undefined Saprotrophic^35^ |
| *Wickerhamomyces* | 0.000 | 0.000 | 0.000 | 0.009 | 0.002 | Saprotrophic | Undefined Saprotrophic^35^ |

**References**

48. Kirk, P. M., Cannon, P. F., Minter, D. W. & Stalpers, J. A. Dictionary of the Fungi: Co-published by Commonwealth Scientific and Industrial Research Organization (CSIRO). *CABI Pub* (2011).

49. Olatinwo, R., So, C. L. & Eberhardt, T. L. Effect of *Acaromyces ingoldii* secondary metabolites on the growth of brown-rot (*Gloeophyllum trabeum*) and white-rot (*Trametes versicolor*) fungi. *Mycobiology*. **47**, 506-511 (2019).

50. Menkis, A., Urbina, H., James, T. Y. & Rosling, A. *Archaeorhizomyces borealis* sp. nov. and a sequence-based classification of related soil fungal species. *Fungal biology*. **118**, 943-955 (2014).

51. Lepelletier, F. *et al*. *Dinomyces arenysensis* gen. et sp. nov. (*Rhizophydiales*, *Dinomycetaceae* fam. nov.), a chytrid infecting marine dinoflagellates. *Protist*. **165**, 230-244 (2014).

52. Boekhout, T., Bai, F. Y. & Nakase, T. *Bullera* Derx (1930). In *The Yeasts* (ed. Kurtzman, C. P., Fell, J. W. & Boekhout, T.) 1623-1659 (Elsevier, 2011).

53. Sun, X. & Guo, L. D. Endophytic fungi VI. *Ciliophora quercus* sp. nov. from China. *Nova Hedwigia*. **85**, 403-406 (2007).

54. Li, D. M., De Hoog, G. S., Saunte, D. L., van den Ende, A. G. & Chen, X. R. *Coniosporium epidermidis* sp. nov., a new species from human skin. *Studies in Mycology*. **61**, 131-136 (2008).

55. Badalyan, S. M. *et al*. New Armenian wood-associated coprinoid mushrooms: *Coprinopsis strossmayeri* and *Coprinellus* aff. *radians*. *Diversity*. **3**, 136-154 (2011).

56. Li, A. H. *et al*. Diversity and phylogeny of basidiomycetous yeasts from plant leaves and soil: Proposal of two new orders, three new families, eight new genera and one hundred and seven new species. *Studies in mycology*. **96**, 17-140 (2020).

57. Soto-Robles, L. V. *et al*. An overview of genes from *Cyberlindnera americana*, a symbiont yeast isolated from the gut of the bark beetle *Dendroctonus rhizophagus* (*Curculionidae*: *Scolytinae*), involved in the detoxification process using genome and transcriptome data. *Frontiers in microbiology*. **10**, 2180 (2019).

58. Gramaje, D. *et al*. Comparative genomic analysis of *Dactylonectria torresensis* strains from grapevine, soil and weed highlights potential mechanisms in pathogenicity and endophytic lifestyle. *Journal of Fungi*. **6**, 255 (2020).

59. Fonseca, Á., Boekhout, T. & Fell, J. W. *Cryptococcus vuillemin* (1901). In *The yeasts* (ed. Kurtzman, C. P., Fell, J. W. & Boekhout, T.) 1661-1737 (Elsevier, 2011).

60. Sampaio, J. P. *Leucosporidium* Fell, Statzell, IL Hunter & Phaff (1969). In *The Yeasts* (ed. Kurtzman, C. P., Fell, J. W. & Boekhout, T.) 1485-1494 (Elsevier, 2011).

61. Liu, X. Z. *et al*. Towards an integrated phylogenetic classification of the *Tremellomycetes*. *Studies in mycology*. **81**, 85-147 (2015).

62. Wuczkowski, M. *et al*. Description of *Holtermanniella* gen. nov., including *Holtermanniella takashimae* sp. nov. and four new combinations, and proposal of the order *Holtermanniales* to accommodate tremellomycetous yeasts of the *Holtermannia* clade. *International journal of systematic and evolutionary microbiology*. **61**, 680-689 (2011).

63. Smith, M. T. & Kurtzman, C. P. *Lipomyces* Lodder & Kreger-van Rij (1952). In *The Yeasts* (ed. Kurtzman, C. P., Fell, J. W. & Boekhout, T.) 545-560 (Elsevier, 2011).

64. Schmidt, S. K. A *Naganishia* in high places: functioning populations or dormant cells from the atmosphere? *Mycology*. **8**, 153-163 (2017).

65. Zhou, Y., Jia, B. S., Zhou, Y. G., Li, A. H. & Xue, L. *Naganishia floricola* sp. nov., a novel basidiomycetous yeast species isolated from flowers of *Sorbaria sorbifolia*. *International Journal of Systematic and Evolutionary Microbiology*. **70**, 4496-4501 (2020).

66. Golzar, H. *et al*. *Neoascochyta* species cause leaf scorch on wheat in Australia. *Australasian plant disease notes*. 14, 1-5 (2019).

67. Chen, C., Verkley, G. J., Sun, G., Groenewald, J. Z. & Crous, P. W. Redefining common endophytes and plant pathogens in *Neofabraea*, *Pezicula*, and related genera. *Fungal Biology*. **120**, 1291-1322 (2016).

68. Sanyal, A., Antony, R., Ganesan, P. & Thamban, M. Metabolic activity and bioweathering properties of yeasts isolated from different supraglacial environments of Antarctica and Himalaya. *Antonie van Leeuwenhoek*. **113**, 2243-2258 (2020).

69. Yurkov, A. M. *et al*. Yeast diversity and species recovery rates from beech forest soils. *Mycological Progress*. **15**, 845-859 (2016).

70. de Gruyter, J. *et al*. Systematic reappraisal of species in *Phoma* section *Paraphoma*, *Pyrenochaeta* and *Pleurophoma*. *Mycologia*. **102**, 1066-1081 (2010).

71. Kurtzman, C. P. & Smith, M. T. *Saccharomycopsis* Schiönning (1903). In *The Yeasts* (ed. Kurtzman, C. P., Fell, J. W. & Boekhout, T.) 751-763 (Elsevier, 2011).

72. Karpov, S. A. *et al*. *Monoblepharidomycetes* diversity includes new parasitic and saprotrophic species with highly intronized rDNA. *Fungal biology*. **121**, 729-741 (2017).

73. Pykälä, J., Kantelinen, A. & Myllys, L. Taxonomy of *Verrucaria* species characterized by large spores, perithecia leaving pits in the rock and a pale thin thallus in Finland. *MycoKeys*. **72**, 43 (2020).
