# Supplementary material for: Diversity, distribution and ecology of fungal communities present in Antarctic lake sediments uncovered by DNA metabarcoding
Source: Sci Rep. 2022 May 19;12:8407. doi: 10.1038/s41598-022-12290-6 (PMC9120451; doi:10.1038/s41598-022-12290-6)
Supplement: Supplementary file 8 — Supplementary Information 8. [file 41598_2022_12290_MOESM8_ESM.docx]

**Diversity, distribution and ecology of fungal communities present in Antarctic lake sediments uncovered by DNA metabarcoding**

Láuren Machado Drumond de Souza, Juan Manuel Lirio, Silvia H. Coria, Fabyano Alvares Cardoso Lopes, Peter Convey, Micheline Carvalho-Silva, Fábio Soares de Oliveira, Carlos Augusto Rosa, Paulo EAS Câmara and Luiz Henrique Rosa

**Supplementary Table S5.** Comparison of diversity indices obtained in studies of Antarctic lake sediment fungal diversity assessed by DNA metabarcoding and traditional culturing methods.

|  |  | **Diversity indices** | | | | | **Citation** |
| --- | --- | --- | --- | --- | --- | --- | --- |
| **Region** | **Lake** | **Number of taxa** | **Number of DNA reads^a^ or isolates^b^** | **Fisher’s-α (diversity)** | **Margalef's (richness)** | **Simpson's (dominance)** |  |
| Elephant Island | Skua | 50 | 33,429^a^ | 21.40 | 9.25 | 0.76 | Current study |
| Deception Island | Soto | 31 | 71,983^a^ | 10.27 | 5.66 | 0.78 |  |
| James Ross | Katerina | 51 | 54,939^a^ | 22.1 | 9.44 | 0.79 |  |
| James Ross | Florencia | 171 | 46,813^a^ | 559.7 | 32.09 | 0.92 |  |
| Elephant Island | Skua | 13 | 64^b^ | 0.79 | 1.11 | 1.22 | Ogaki et al.^9^ |
| Deception Island | Soto | 6 | 20^b^ | 0.61 | 0.47 | 0.54 |  |
|  | Deception B | 0 | 0^b^ | 0 | 0 | 0 | Ogaki et al.^9^ |
|  | Kroner | 3 | 9^b^ | 0.60 | 0.29 | 0.39 |  |
| Fildes Peninsula, King George Island | North | 41 | 122^b^ | 5.25 | 4.22 | 0.94 |  |
|  | Central | 15 | 43^b^ | 2.06 | 1.75 | 0.86 | Ogaki et al.^4^ |
|  | South | 22 | 95^b^ | 2.51 | 2.17 | 0.8 |  |
| Admiralty Bay, King George Island | Punta Hennequin | 8 | 33^b^ | 0.81 | 0.72 | 0.78 |  |
|  | Wanda A | 10 | 20^b^ | 0.81 | 1.16 | 1.37 | Ogaki et al.^9^ |
|  | Wanda B | 5 | 10^b^ | 0.63 | 0.59 | 0.70 |  |
| Penguin Island | Petrel | 15 | 39^b^ | 0.82 | 1.58 | 1.81 | Ogaki et al.^9^ |
| Hope Bay | Boeckella (top) | 114 | 36,471^a^ | 41.7 | 17.66 | 0.89 |  |
|  | Boeckella (middle) | 55 | 26,582^a^ | 14.74 | 8.44 | 0.88 | Rosa et al.^12^ |
|  | Boeckella (base) | 146 | 30,088^a^ | 61.44 | 22.67 | 0.89 |  |
| Vega Island | Copépodo | 161 | 223,679^a^ | 16.97 | 12.99 | 0.81 |  |
|  | Esmeralda | 224 | 640,902^a^ | 21.77 | 21.77 | 0.74 | Ogaki et al.^11^ |
|  | Pan Negro | 116 | 243,518^a^ | 11.66 | 9.27 | 0.66 |  |
| Fildes Peninsula, King George Island | North | 41 | 122^b^ | 5.25 | 4.22 | 0.94 |  |
|  | Central | 15 | 43^b^ | 2.06 | 1.75 | 0.86 | Ogaki et al.^4^ |
|  | South | 22 | 95^b^ | 2.51 | 2.17 | 0.8 |  |
| Admiralty Bay, King George Island | Punta Hennequin | 8 | 33^b^ | 0.81 | 0.72 | 0.78 |  |
|  | Wanda A | 10 | 20^b^ | 0.81 | 1.16 | 1.37 | Ogaki et al.^9^ |
|  | Wanda B | 5 | 10^b^ | 0.63 | 0.59 | 0.70 |  |
| Penguin Island | Petrel Lake | 15 | 39^b^ | 0.82 | 1.58 | 1.81 | Ogaki et al.^9^ |
| Deception Island | Deception A | 6 | 20^b^ | 0.61 | 0.47 | 0.54 |  |
|  | Deception B | 0 | 0^b^ | 0 | 0 | 0 | Ogaki et al.^9^ |
|  | Kroner | 3 | 9^b^ | 0.60 | 0.29 | 0.39 |  |
